# A Systematic Review of Methodological Approaches to SARS-CoV-2 Wastewater Surveillance

**DOI:** 10.3390/v18020205

**Published:** 2026-02-04

**Authors:** György Deák, Laura Lupu, Raluca Prangate

**Affiliations:** 1 National Institute for Research and Development in Environmental Protection, Splaiul Independenţei 294, 060031 Bucharest, Romania; dkrcontrol@yahoo.com (G.D.); laura.lupu@incdpm.ro (L.L.); 2 Doctoral School of Biotechnical Systems Engineering, National University of Science and Technology POLITEHNICA of Bucharest, Splaiul Independenței 313, 060042 Bucharest, Romania

**Keywords:** COVID-19, risk transmission, SARS-CoV-2, surveillance, wastewater, wastewater-based epidemiology

## Abstract

Following the COVID-19 pandemic, researchers have increasingly focused on monitoring the spread of the virus and improving methods to detect changes in the SARS-CoV-2 genome. Although clinical surveillance provides direct and reliable results, it has limited applicability. Wastewater-based epidemiology (WBE) has therefore emerged as a valuable, non-invasive complementary tool for disease surveillance. It provides a comprehensive picture of virus circulation in a population, including asymptomatic individuals and those who do not seek healthcare. In addition, it facilitates early detection of outbreaks and the collection of epidemiologic data at the community level. However, WBE also presents technical challenges, including variations in sampling and testing protocols, the presence of inhibitors that affect viral RNA extraction, and the need for standardised procedures between studies. These challenges should be addressed for possible future infectious disease outbreaks. One of the challenges facing researchers was to develop efficient methods that could overcome the extraction and detection problems related to inhibitors present in wastewater. To this aim, this systematic review highlights the potential use of WBE, the variety of techniques, and the most effective methods for the detection and quantification of SARS-CoV-2 in wastewater samples. A reproducible electronic search of the literature was conducted in the Web of Science (WoS) and PubMed databases for articles published between 2020 and 2024. Our search revealed that the majority of observed WBE applications emphasised a correlation between SARS-CoV-2 RNA concentration trends in wastewater and epidemiological data. Another relevant issue that the articles often discussed and compared was the techniques used in different steps of sample processing, such as sample collection, concentration and detection, hence the lack of standardised procedures. This paper provides a framework regarding previous research on WBE to gain a better understanding that will lead to functional solutions.

## 1. Chronology of Events Since the First Cases of COVID-19 Were Identified to the Epidemiological Monitoring of Wastewater

At the end of 2019, patients diagnosed with pneumonia with no known aetiologic agent were identified in multiple health facilities in Wuhan, Hubei Province, China [1]. According to epidemiologic investigations, atypical pneumonia was correlated with the Huanan market [2], which is known for its trade of seafood and other live animals [3]. In early January 2020, scientists identified a novel coronavirus associated with severe viral pneumonia as the causative pathogen, which was initially named nCoV-2019 [1]. Approximately one month after the first reported case of infection with the new coronavirus, the viral RNA was identified using advanced sequencing techniques [4]. On 12 January 2020, China published the genome of the new coronavirus. The spread of the novel coronavirus continued at an alarming rate and was facilitated by travel, overcrowded events and asymptomatic individuals. On 30 January 2020, the World Health Organisation (WHO) declared the outbreak a Public Health Emergency of International Importance [5], and on 11 February 2020, the new disease was given the name COVID-19 [6]. On February 11, the International Committee on Taxonomy of Viruses (ICTV) named the virus SARS-CoV-2 [7]. On 11 March 2023, the WHO officially declared the COVID-19 crisis a global pandemic [8] after SARS-CoV-2 spread to more than 114 countries [9].

The complete genetic code of SARS-CoV-2 was obtained through next-generation sequencing (NGS). Based on the genetic characteristics and phylogenetic relationships, the virus responsible for the COVID-19 pandemic was introduced into the virus family tree as follows: kingdom—Orthornavirae, phylum—Pisuviricota, class—Pisoniviricetes, order—Nidovirales, family—Coronaviridae, subfamily—Orthocoronavirinae, genus—Betacoronavirus, and subgenus—Sarbecovirus [7,10].

The SARS-CoV-2 viral genome is a single linear strand of positive-sense RNA that is approximately 29.9 kilobases (kb) in length. Its genetic code consists of open reading frames (ORFs) that encode 27 proteins and are classified into 3 categories: structural proteins, nonstructural proteins and accessory proteins. The main structural proteins are the spike protein (S), nucleocapsid protein (N), membrane protein (M) and envelope protein (E) [11]. Nonstructural proteins are produced by the translation and processing of the ORF1a and ORF1b polyproteins. After being translated into a large polyprotein, viral proteases cleave the polyprotein to form 16 nonstructural proteins (Nsp1–Nsp16). SARS-CoV-2 also expresses the following accessory proteins: ORF3a, ORF6, ORF7a, ORF7b, ORF8, ORF9b, and ORF10. Although these proteins are not essential for viral replication, they may influence pathogenicity and the interaction with the host immune system [12].

The urgent need to monitor the spread of the virus around the world led researchers to develop effective methods to detect the SARS-CoV-2 viral RNA from wastewater samples collected from various pandemic-affected communities [13]. Nevertheless, data on the occurrence of the virus is derived from its detection by molecular methods, which identify fragments of viral RNA rather than viable, infectious viruses [14]. The concept of wastewater surveillance has been previously used to monitor other pathogens, such as poliovirus [15]. Multiple groups of researchers from different states have initiated studies to detect SARS-CoV-2 viral RNA in wastewater samples to support individual clinical epidemiologic surveillance. The first positive results were reported in the summer of 2020 [16]. Moreover, in March 2021, the European Commission published the “Commission Recommendation 2021/472 of 17 March 2021, on a common approach to establish a systematic surveillance of SARS-CoV-2 and its variants in European Union wastewater” [17]. Soon after, many countries in the European Union (EU) started to implement strategies for monitoring SARS-CoV-2 and its variants in wastewater [18,19].

Based on the results obtained by implementing these monitoring systems, epidemiologic surveillance of wastewater has become an essential monitoring tool that is complementary to clinical data [20]. Surveillance of pathogens of concern in wastewater has made early detection possible before the onset of symptoms in the community, thus providing time for authorities to take action [13]. One of the advantages of such a method is that it does not cost as much as clinical surveillance does, allowing the detection of infections in asymptomatic individuals who would not have been clinically tested in the absence of symptoms but who contributed to the spread of the virus [21,22]. The overview provided by surveillance of pathogens in wastewater has produced information regarding the effectiveness of measures implemented by public health authorities [13,23,24]. These developments highlighted the need to urgently investigate the environmental pathways of SARS-CoV-2, with wastewater quickly becoming a key area of focus [25].

Considering this background, the rapid emergence and global spread of SARS-CoV-2, along with its persistence and potential transmission risks in aquatic environments, wastewater-based epidemiology (WBE) has emerged as a crucial complementary tool for monitoring viral circulation. This research makes a contribution to the literature by providing a comprehensive and integrative analysis of WBE in the context of SARS-CoV-2. This systematic review aims to highlight the potential application of WBE, the available techniques, and the most effective methods for determining viral loads and circulating variants of SARS-CoV-2 in wastewater samples. Specifically, the objectives are to identify the most commonly used detection strategies and sequencing platforms. The two-database, multidimensional approach employed in this study combined evidence from PubMed and Web of Science (WoS), with strict inclusion criteria applied to peer-reviewed studies demonstrating strong methodological relevance. This enables a more nuanced understanding of the public health implications of SARS-CoV-2 in wastewater. This integrative perspective aims to inform future guidelines for monitoring, risk assessment, and prevention.

## 2. Materials and Methods

**Figure 1:**
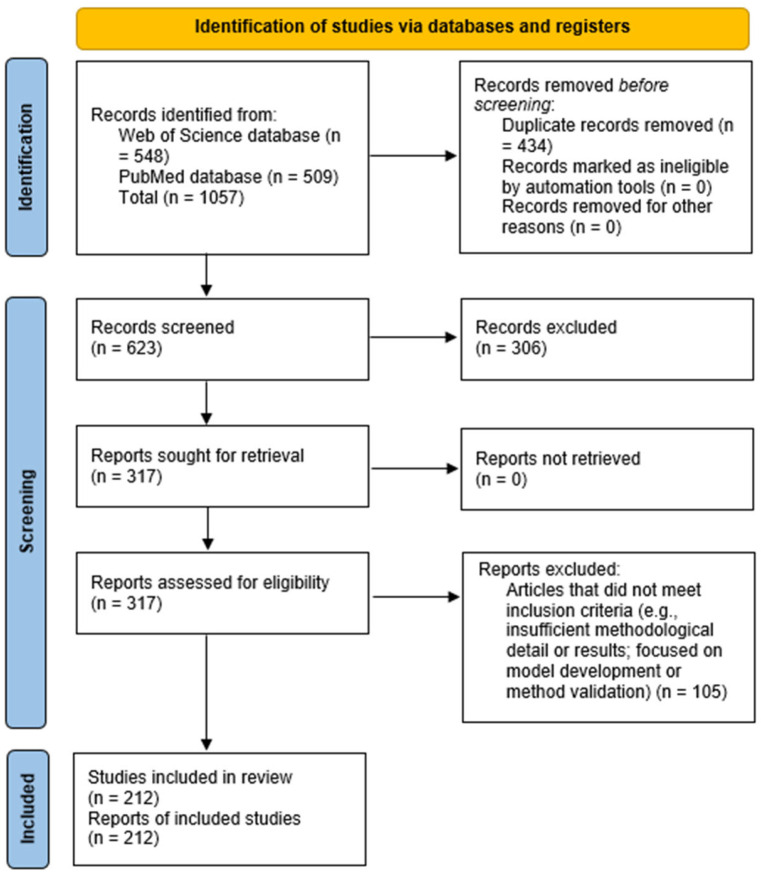
Preferred Reporting Items for Systematic Reviews and Meta-Analyses (PRISMA) flow diagram illustrating the process of selecting publications for evidence-based research [26].

In accordance with the Preferred Reporting Items for Systematic Reviews and Meta-Analyses (PRISMA) guidelines [26], a systematic literature review was conducted through an electronic search of the WoS and PubMed databases. The search strategy targeted peer-reviewed articles published in English between January 2020 and 24 October 2024. A combination of keywords related to ‘SARS-CoV-2′, ‘wastewater’, ‘surveillance’, ‘quantification’ and ‘environmental monitoring’ was used. Details of the search terms used and the number of records retrieved are presented in Table 1. In view of the substantial volume of publications retrieved from a single search query, the filtering steps outlined in Table 1 were applied in order to reduce the initial dataset and to obtain a relevant and manageable set of articles for analysis. Reviews and preprints were excluded on the basis of their automatic exclusion. This systematic review was not registered, and a protocol was not prepared. A total of 548 articles were downloaded from the WoS database, and 509 articles from PubMed, and metadata from both databases, including title, abstract, keywords, authors, and reference lists, were extracted for further examination. Following a thorough review, it was determined that 434 peer-reviewed articles were duplicates and were therefore excluded from further consideration. The selection of articles was performed manually by two reviewers, with the following inclusion criteria applied: The following methods are to be employed for the early detection of outbreaks: firstly, the use of surveillance methods, secondly, the monitoring of viral concentration trends in wastewater, and thirdly, the assessment of the prevalence of SARS-CoV-2 infections in monitored communities. The diversity of circulating viral variants was also taken into account. Initial screening was performed based on the title and abstract, followed by a full-text assessment. Articles were excluded if they focused on topics unrelated to the objective of this review, such as clinical diagnosis or treatment of COVID-19, study of other viral pathogens, or analysis in matrices other than wastewater (e.g., soil, leachate, air). Following the selection process, 212 articles were identified as meeting the eligibility criteria and were included in the final review. However, as a limitation, the number of studies was influenced by the inclusion criteria and could not contain the entire scientific corpus. The selection steps are presented in Figure 1, in accordance with the PRISMA guideline.

**Figure 2:**
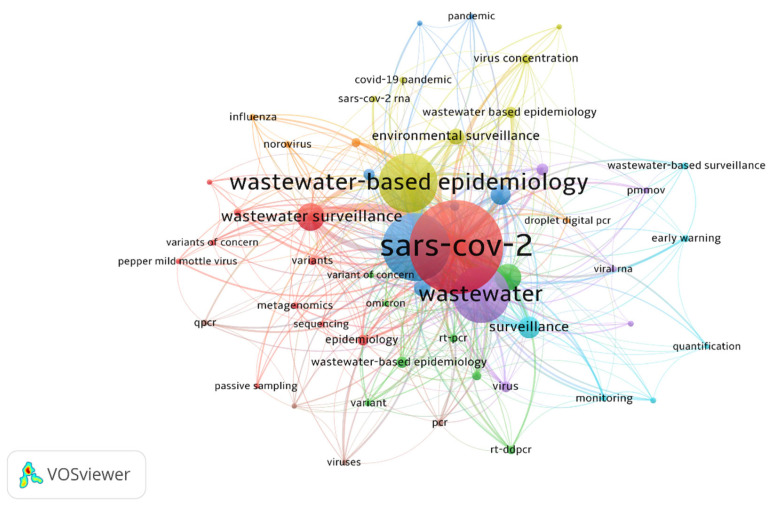
Keyword network co-occurrence of articles using “VOSviewer version 1.6.20, 2023” [27]. The keywords network visualization shows the items by labels and spheres. The size of the tag and sphere of an item are determined by the importance of the number of keyword appearances in the title of the analyzed articles. The size variation in lines represents how strong are the links between items.

In order to explore thematic patterns within the literature, a keyword co-occurrence network analysis was performed using VOSviewer software (version 1.6.20, 2023) [27]. The application of a minimum occurrence threshold of five resulted in the inclusion of 53 keywords out of 908 in the analysis. The keywords were then grouped into eight distinct clusters: Cluster 1 (11 terms, red), Cluster 2 (8, green), Cluster 3 (7, blue), Cluster 4 (7, yellow), Cluster 5 (6, purple), Cluster 6 (6, turquoise), Cluster 7 (4, orange), and Cluster 8 (4, brown), as illustrated in Figure 2.

## 3. Results and Discussion

### 3.1. Overview of the General Characteristics in the Included Studies

In the SARS-CoV-2 wastewater surveillance process, there are many variables affecting the coherence and quality of findings, such as sampling strategies, ambient conditions, concentration methods of viral RNA, and statistical interpretation strategies that are selected [28]. Numerous methods have been developed for detecting and quantifying SARS-CoV-2 viral RNA in wastewater. In most studies, the main steps of the workflow were the addition of a matrix recovery control, removal of solid particles, determination of the viral RNA concentration, extraction, detection and quantification [29]. Next-generation genome sequencing can also identify new circulating strains of SARS-CoV-2 [30]. The detection of viruses in wastewater faces limitations such as the large amount of water that needs to be processed and its matrix, which is rich in organic matter and microorganisms [31].

The recovery of viruses from water samples requires concentrating them from a large volume of water into a much smaller volume to make viral detection possible [32]. Efficient concentration methods must be used before extraction and detection to obtain relevant results [33]. Electronegative and electropositive membranes are often used to concentrate enteric viruses from treated and untreated water samples. Another method that uses membranes is ultrafiltration, which is based on size exclusion [34]. Other concentration techniques include polyethylene glycol (PEG) precipitation, centrifugation, and skim milk flocculation [35,36]. The previously mentioned methods have been used for different types of water (groundwater, wastewater, drinking water, etc.).

Since the beginning of the pandemic, the polymerase chain reaction (PCR) based method has been used to detect and quantify SARS-CoV-2, as it offers sensitivity, specificity and quick detection [37]. This technique is also adaptable depending on the virus type, as probes and primers can be modified according to the needs of the study [38]. The method most widely used for the quantification of viruses from sewage is quantitative reverse transcription PCR (qRT-PCR). This method detects a small segment of the viral genome, facilitating the sensitive and accurate identification of genetic regions [39]. However, it is susceptible to inhibition due to contaminants in wastewater and variability in RNA extraction efficiency [40]. Another type of PCR used for quantification is the real-time reverse transcription-PCR (RT-PCR) method [41]. PCR assays target the following regions of SARS-CoV-2: ORFs, N, E, S and genes encoding RNA-dependent polymerases. For wastewater monitoring, N regions (N1, N2 or N3) are the most frequently used genes [37].

For a better understanding of SARS-CoV-2 behaviour and circulating variants, some studies have taken additional steps. NGS facilitates whole-genome sequencing of SARS-CoV-2, identifying variants and tracking evolution [42]. This method provides high specificity and can simultaneously detect multiple viral variants, making it invaluable for monitoring emerging variants. However, NGS requires extensive computational resources, longer processing times and higher costs, which limit its widespread use in real-time surveillance. Additionally, NGS provides relevant information that can help advance the development of vaccines, as well as diagnostic tests, and provides insights into the phylogeny of the virus [43]. Analysis of sequencing data from wastewater samples requires the use of bioinformatics tools for processing the raw data and assembling viral genomes. To accomplish this, a number of key computational approaches were implemented. Global Initiative on Sharing All Influenza Data (GISAID) for geographical distribution of variants, Nextstrain for phylogenetic tree visualisation and variant annotation, and Phylogenetic Assignment of Named Global Outbreak Lineages (Pangolin) for lineage assignment are just a few of these tools. However, many others exist that help in evolutionary and epidemiological analysis, such as BlueDot, PAUP, Fast-tree, CovidPhy, Covidex, etc. [44].

**Figure 3:**
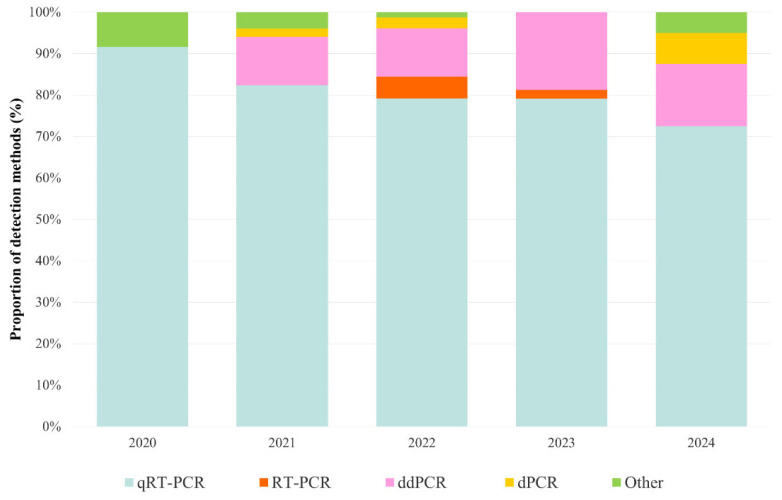
Trends in the usage rates of different detection methodologies.

Supplementary Table S1 summarises the research conducted during the SARS-CoV-2 pandemic in different countries, including data about the methods used for detection and monitoring. In most studies, samples were collected from wastewater treatment plants or from the sewage network. The volume of the collection varied greatly between studies, but 500 and 1000 mL were the most common choices [19,24,45,46,47,48,49,50]. Larger volumes, such as 10 L, were also collected [51]. For instance, the study of Lombardi [45] collected 500 mL, the study of Sousa [52] used 200 mL, the study of Toledo [53] used 100 mL, Brumfield [54] collected 60 mL, and Deák [19] used 1000 mL [19,45,52,53,54]. Sample concentration methods ranged from simple homogenisation to the most frequently applied, PEG precipitation. The remaining studies chose to use centrifugation, ultracentrifugation, ultrafiltration, passage through electronegative membranes, concentrators to affinity capture magnetic hydrogel particles or aluminium-driven flocculation. For the extraction step, various kits were used, such as the QIAamp^®^ Viral RNA Mini Kit (Qiagen, Hilden, Germany), the AllPrep PowerViral DNA/RNA Kit (Qiagen, Hilden, Germany), the MagMAX^™^ Viral/Pathogen Nucleic Acid Isolation Kit (Thermo Fisher Scientific, MA, USA), the Promega Wastewater Large-Volume RNA Capture Kit (Promega, Madison, WI, USA), MagMax CORE Nucleic Acid Purification Kit (Thermo Fisher Scientific, MA, USA), RNeasy PowerMicrobiome Kit (Qiagen, Hilden, Germany) and others [19,48,49,50,52,53,54,55]. The presence of SARS-CoV-2 in wastewater has been analysed using different techniques of RNA amplification and quantification. Among the techniques used in the reviewed articles, we mention Nested RT-PCR, qRT-PCR, RT-PCR, droplet digital PCR (ddPCR) and digital PCR (dPCR). The main amplification method that was used in the studies from Supplementary Table S1 is qRT-PCR. Figure 3 shows the trends in detection methods from 2020 to 2024. The most frequently used method was qRT-PCR across the years, followed by ddPCR, dPCR and RT-PCR. Other methods used across studies were: Loop-Mediated Isothermal Amplification (LAMP), Volcano 2nd Generation qPCR (V2G-qPCR), solid digital PCR (sdPCR), Single Nucleotide Polymorphism PCR (SNP-PCR) and Nested PCR. Since 2021, other technologies such as dPCR and ddPCR have become more widely used. The primers for the N1, N2 and E genes were predominant in the PCR-based SARS-CoV-2 detection assays. In most cases, the primers were provided by the Centres for Disease Control and Prevention (CDC) for N1, N2, and N3 or synthesised for the E gene according to Corman’s design [29,56]. However, a variety of primers were used, including those targeting the RdRP, ORF1ab and S genes [57,58,59,60]. The papers provided quantitative data on the concentrations obtained in genome copies per litre (G.C./L) or genome copies per millilitre (G.C./mL), limit of detection (LOD), limit of quantification (LOQ) values or the positivity rate. Among the research papers, the results varied depending on the concentration method, detection assay and water matrix from the site of sample collection. Generally, wastewater samples presented relatively high concentrations, ranging between 10^1^ and 10^17^ G.C./L. Besides wastewater studies, a few studies monitored river water. For example, two research papers that collected samples from rivers located in Serbia and Japan obtained values between 5.97 × 10^3^ and 2.8 × 10^5^ G.C./L [61,62]. In addition, research has shown a correlation between positive wastewater samples and clinical cases. Claro [63] reported that RNA was present in wastewater at the same time recurring local outbreaks of COVID-19 in Brazil started to be recorded [63]. Chai [64] also observed a strong correlation between weekly wastewater data and the positive rate of COVID-19 in sentinel hospitals in China and noted that SARS-CoV-2 was present in wastewater before clinical cases were reported [64]. In the study conducted by Martins [65], a similar correlation was observed: the virus could be detected in wastewater 5 days (average) before new positive cases were reported [65].

A range of sequencing platforms has been utilised to detect and characterise SARS-CoV-2 in wastewater samples. The studies that performed sequencing used NGS platforms such as NovaSeq 6000, NextSeq 1000, NextSeq 2000, NextSeq 500, MiniSeq, MiSeq, HiSeq, Ion Torrent, GridION, MinION and ATOPlex V3.1. The Sanger method has also been used to confirm the specificity of qRT-PCR assays by sequencing only the gene of interest from the SARS-CoV-2 genome. With these technologies, different primer panels have been used, such as ARCTIC V3, V3.1, V4, V4.1, V5.3.2 for Illumina and Oxford Nanopore platforms and Ion AmpliSeq SARS-CoV-2 for Ion Torrent. Among these primer panels, some have been found to have improved genome coverage, as in the case of the ARCTIC V4 primer, which was reported to be better than previous ARCTIC primers [66]. A comparative study of the ARCTIC and Ion AmpliSeq primers revealed that both exhibited comparable efficacy in terms of sensitivity, single-nucleotide variant (SNV) calling, and lineage assignment [67]. The articles listed in Supplementary Table S1, which involved NGS emphasised the importance of monitoring the strains present in the community. All variants (Alpha, Delta and Omicron) and their subvariants have been repeatedly detected through sequencing. Jahn [68] reported the identification of Alpha (B.1.1.7) and Delta (B.1.617) variants before clinical detection in some of the monitored locations in Switzerland. Additionally, they noted that if the sample size is larger, WBE detection has an advantage over clinical case reports. Research that monitored the genetic diversity of SARS-CoV-2 in the Netherlands and Belgium revealed several novel mutations in its genome and identified prevalent clades (19A, 20A, and 20B) from 25 March to 3 June 2020 [69]. Similarly, Hillary [70] assessed the genetic diversity of SARS-CoV-2 and discovered 702 unique single-nucleotide polymorphism (SNP) sites and 267 indels across 84 samples [70]. For variant calling, articles chose different tools such as VarScan, Unipro Ugene, Genome Analysis Toolkit software (GATK) (V.4.2.0.0), and Integrated Genomics Viewer (IGV). The preferred data-sharing platform in these studies was GISAID [69,71,72,73]. However, the platform was used for other purposes as well such as mutation analysis [74]. All these studies highlight the importance of wastewater sequencing, as it helps detect prevalent strains before clinical case reporting, monitor circulating variants and identify regional mutations.

### 3.2. Geographical Characteristics and Regional Differences in Global WBE Research

**Figure 4:**
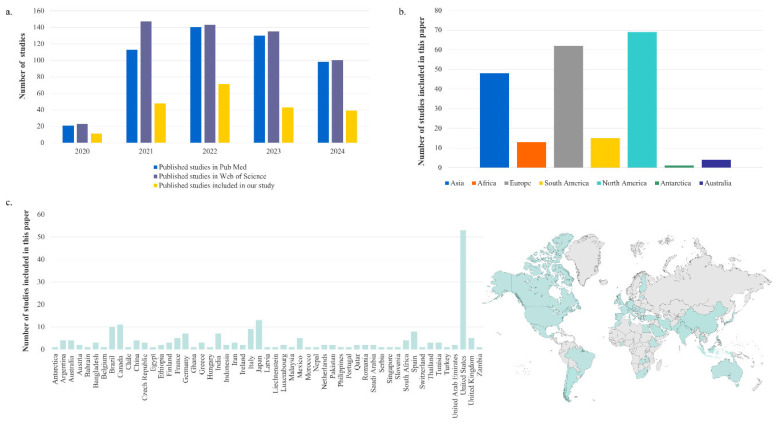
Bibliometric analysis and geographical distribution of literature: (**a**) Annual evolution of WBE research, comparing the total number of articles retrieved from PubMed and Web of Science (WoS) with the final selection of studies included in this study (2020–2024); (**b**) Distribution of included studies by continent; (**c**) Geographic distribution of studies by country and the corresponding world map.

To provide a more comprehensive overview of our findings, Figure 4 represents a comparison between the number of included studies and those found in the databases, the distribution of selected articles by continent, and the map with the geographic coverage. As illustrated in Figure 4a, the number of studies published in 2021 exceeded 140, with a similar trend observed in subsequent years, with 2022 and 2023 also experiencing similar numbers. However, the number of studies decreased in 2024.

Figure 4b, c illustrate that North America leads with 69 studies; following Europe with 62 studies, Asia with 48, South America with 15, Africa with 13, Australia with 4 and Antarctica with 1. In Europe, the countries that have conducted the most studies are Italy (9), Spain (8), Germany (7), France (5 studies), and the United Kingdom (5). The majority of studies in North America were conducted in the United States (53), followed by Canada (11) and Mexico (5). Within the Asian continent, Japan (13) and India (7) exhibited the highest number of studies, while in South America, Brazil (10) emerged as the predominant country. Other nations such as Malaysia, Nepal, Morocco, the Philippines, and Turkey each published one article. This issue may be attributed to the influence of language bias and the disparities between English-speaking and non-English-speaking regions [75]. As it was observed before, WBE is more available in developed countries than in developing countries [76,77]. These results suggest that financial resources and infrastructure play an important role in research activities and are often conducted in urban areas with high population sizes [78]. However, as these numbers are based only on data that could be retrieved from Supplementary Table S1, they do not represent an exhaustive analysis of all SARS-CoV-2 surveillance studies. Given the importance of the subject, many other countries took measures to better understand the presence of SARS-CoV-2 in wastewater.

### 3.3. Normalisation Strategies for SARS-CoV-2 Data in WBE

Normalisation data is a necessary measurement in WBE, as it allows for the correction and addressing of variations resulting from dilution and faecal discharge [79]. The factors affecting SARS-CoV-2 concentrations include processing techniques, contributing populations, wastewater dilution, faecal shedding rates and wastewater composition. Therefore, normalisation methods can use recovery control techniques, or biomarker and environmental normalisation techniques, which include markers for faecal content, population served and environmental conditions. While recovery controls address technical variability, biomarker and environmental normalisation are meant to account for sample variability [80]. Several surrogate viruses have been used as process controls: bovine respiratory syncytial virus (BRSV), bovine coronavirus (BCoV), murine hepatitis virus (MHV), bacteriophage Phi6, human coronavirus OC43, human coronavirus HCoV-E, F-specific RNA phages [81,82,83]. These recovery control viruses have the role of improving the accuracy of viral load estimates in wastewater monitoring [84,85].

The composition of wastewater varies due to sewer inflow, runoff, industrial discharges and extraneous waters. For this reason, flow rate normalisation is required to account for the dilution rate [86,87]. However, when the flow rate is unreliable, faecal and population biomarkers can improve the interpretation of SARS-CoV-2 measurements by addressing additional sources of variability. For instance, Langeveld [87] demonstrated that normalisation using crAssphage and/or electrical conductivity can complement flow rate [87]. The most widely used indicators of human faecal contamination in SARS-CoV-2 RNA detection are crAssphage and Pepper Mild Mottle Virus (PMMoV) due to their frequent presence in faeces [88,89]. D’Aoust’s [90] compared three biomarker gene regions for faecal normalisation: human-specific HF183 Bacteroides 16S rRNA, human eukaryotic 18S rRNA, and PMMoV. The results showed that PMMoV was more consistent and variable, making it more suitable for normalisation [90]. Another relevant aspect for interpreting viral loads and understanding the spread of viruses in communities is the presence of population biomarkers in wastewater [91]. Hsu [92] compared different normalisation coefficients among CAF, PARA, PMMoV and 5-HIAA. The results of the direct normalisation (C1(i)) approach showed that CAF performed better, with lower variation and higher precision, followed by PARA, 5-HIAA and PMMoV. When the indirect normalisation approach was used to calculate normalisation coefficient 2 (C2(i)), it was observed that, due to its better population indicators with higher accuracy, lower variance and higher temporal consistency, PARA is a more reliable population biomarker than PMMoV. The study also reported that PARA formed more stable correlations with the population, enabling better normalisation of SARS-CoV-2 per capita [92]. These population biomarkers play an important role in normalising RNA concentrations in wastewater, but further research is needed to complete and standardise the methods to normalise the quantification of SARS-CoV-2 RNA concentration in wastewater [92,93].

Several normalisation strategies have been described for the detection of SARS-CoV-2 in wastewater, in order to account for the various sources of variability in the water matrix. As water dilution and population changes are the most prominent sources of variability, measurements such as flow rate and biochemical measurements can help address these issues [89]. Flow rate normalisation accounts for dilution effects, enabling the conversion of viral concentrations into daily loads [86]. Population-based normalisation provides further context for viral signals by adjusting for the size of the contributing population, facilitating comparisons across catchments and aligning with epidemiological indicators [94]. Faecal biomarkers such as PMMoV and crAssphage are also useful for reflecting human faecal input and correcting for fluctuations in wastewater composition, especially when flow or population data is unreliable [87,89]. However, for example, flow rate does not fully capture variations in human contribution, as wastewater alone can fluctuate independently. This limitation can be addressed by using complementary methods such as faecal markers (e.g., PMMoV and crAssphage) or electrical conductivity [87]. It should be noted, though, that the presence of PMMoV depends greatly on the season and local food sources, and that the shedding of crAssphage varies from person to person and should only be studied in populations of over 5000 people [89]. Similar inconsistencies can also be found in population biomarkers, such as CAF, PAR and 5-HIAA, since these are affected by individual excretion rates and seasonal changes [80]. Therefore, normalisation strategies should employ a combined approach to address the multiple sources of variability.

### 3.4. Comparative Analysis of WBE Pipelines

The WBE workflow includes viral concentration, nucleic acid extraction, molecular detection and sequencing to enable more in-depth analysis. Since each step can introduce variability and influence sensitivity, recovery efficiency, and quantitative accuracy, numerous studies have compared the efficiency of different concentration, amplification, and sequencing methods.

A recent study compared various techniques for recovering SARS-CoV-2 from wastewater, including ultrafiltration, PEG precipitation, aluminium chloride (AlCl_3_) flocculation, and skim milk flocculation. RNA from COVID-19 patients and Pseudomonas phage φ6 were used as a control. The results indicated that there were significant differences among the various methods employed in terms of recovery efficiency, with ultrafiltration and AlCl3 precipitation being the most promising with 42.0% and 30.0% recovery rates, respectively. The E gene was the sole viral marker consistently detected across all the samples being studied [95]. Barril [96] also compared several concentration techniques and found that PEG precipitation and polyaluminium chloride (PAC) flocculation had the highest recovery efficiency (62.2% and 45.0%, respectively) [96]. Flood [97] compared the recovery efficiency of Phi6 following the application of two ultrafiltration methods and PEG concentration to different types of wastewater. The results showed that PEG precipitation achieved a higher recovery efficiency (mean of 22.19% to 51.47%) than the two ultrafiltration methods (mean of 2.6% to 11.6%). Although direct quantitative comparisons are limited by differences in methodology, both Barril [96] and Flood [97] reported that PEG precipitation had higher recovery efficiencies [96,97]. Pérez-Cataluña [98] tested the efficiency of an aluminium-based adsorption-precipitation method using PEG, seeding the wastewater with gamma-irradiated SARS-CoV-2, porcine epidemic diarrhoea virus (PEDV) and Mengovirus (MgV). Nevertheless, the study concluded that there are no significant differences between the two concentration methods, with factors such as the extraction method and molecular target also influencing the outcome [98].

Quantification of SARS-CoV-2 RNA in wastewater is an important stage in COVID-19 pandemic surveillance that shows high variability due to factors such as analytical uncertainty of the analysis, variation in the total amount of human faeces in wastewater and other aspects responsible for noise in measurement [13,59,60,61]. One study compared qRT-PCR and RT-ddPCR detection of SARS-CoV-2 in wastewater samples, targeting the N1, N2 and E genetic markers. Results showed that RT-ddPCR was more sensitive and accurate than qRT-PCR for the detection of both SARS-CoV-2 genetic markers. RT-ddPCR consistently detected all three markers, whereas qRT-PCR reported levels of the E gene at or below the detection limit [97]. Additionally, seeding experiments with SARS-CoV-2 in wastewater revealed that the N1 and N2 dPCR assays yielded positive results despite qPCR showing negative results for the two genes in low concentration seeding scenario. This could be due to the ability of dPCR to split the sample into numerous reactions, thus facilitating absolute quantification without the need for standard curves. The study by Ahmed [40] compared the two methods and reported positivity rates of 27.1% for N1 and 18.8% for N2 when using RT-dPCR for the combined pellet and eluate, compared to 5.20% for N1 and 0% for N2 when using qRT-PCR [40]. The technology of dPCR has proven to be particularly efficient in analysing low-concentration samples, a feature that makes it suitable for longitudinal surveillance scenarios where viral loads may be subject to variability. However, dPCR is associated with higher costs and time requirements, and the need for dedicated equipment restricts its potential for large-scale use [37]. D’Aoust [90] also compared the performance of qRT-PCR and RT-ddPCR in detecting SARS-CoV-2 in post-grit solids (PGS) and primary clarified sludge (PCS) during periods of low incidence. For both the N1 and N2 genes, the study reported an LOD of two copies per reaction for qPCR and five copies per reaction for RT-ddPCR. In comparison, Ahmed [40] study, the RT-ddPCR showed a higher inhibition than qRT-PCR when used on pegged sludge matrices [40,90]. Länsivaara [99] found the LOD of ddPCR to be lower (0.06 G.C./µL) compared to other qRT-PCR gene assays (18.4, 19.9, 77.6 and 80.7 G.C./µL for TaqMan N1, N2, QuantiTect N1 and N2 assays). Despite this, the study found that qRT-PCR results were more closely correlated with the incidence of the virus [99]. As the number of comparative studies between different PCR-based methods remains limited, there is a necessity for further research in this area.

In recent years, other methods of detecting the presence of SARS-CoV-2 in wastewater have been explored. One such method uses RT-LAMP on microfluidic chips, allowing for quick detection with or without prior sample concentration. This method could offer a cheaper, faster alternative to viral RNA detection [100]. Electrochemical biosensors that rely on bioreceptors have also attracted attention as they have previously been used to identify Ebola, Zika and human immunodeficiency viruses. These sensors can be made of molecularly imprinted polymers, aptamers, or antibodies, and can successfully detect SARS-CoV-2 in aqueous environments. Such techniques could provide an alternative to classical PCR detection in wastewater from low-income regions and reduce sample preparation time [101].

Compared to molecular detection methods, sequencing techniques present additional challenges when used on wastewater samples. The success of sequencing depends on factors such as library preparation, viral RNA concentration, and amplification efficiency. Sequencing coverage is further impacted by RNA fragmentation, PCR inhibitors and amplicon dropout [102,103]. To address issues relating to the low viral load, Paden [104] employed a specific set of primers to generate nested, tiling amplicons [104]. For amplicon dropout, constant updating of the amplicon panel is required as new mutations are identified, or primer binding sites will result in uneven sequencing coverage [105]. Furthermore, tools developed for clinical samples, such as Pangolin and UShER15, are not suitable for defining mixed viral lineages from wastewater samples. This issue was addressed by Karthikeyan [106]) with the proposal of Freyja, a tool that uses SNVs as a barcode to estimate the relative abundance of virus lineages in a mixed sample, rather than focusing on complete genomes. The study reported that this method could detect lineages before clinical detection [106].

To facilitate the practical application of these techniques, Table 2 presents a workflow identifying key decision points for WBE implementation tailored to specific surveillance objectives. Additionally, Table 3 provides further details on the advantages and disadvantages of commonly used quantification methods and sequencing platforms, as well as their specificity, feasibility and sensitivity.

### 3.5. Correlation Between Monitoring of SARS-CoV-2 in Wastewater and Clinical Data Using Predictive Models

Many studies support the correlation between concentrations of SARS-CoV-2 RNA in wastewaters and clinical case numbers, developing predictive models using advanced statistical methodologies [107,108,109]. In Table 4, a summary of modelling techniques used in SARS-CoV-2 WBE has been made. These models often combine techniques such as time-series analysis, machine learning algorithms, and regression models, including non-parametric approaches using Spearman and Pearson correlations, to forecast future case trends. Of the 24 analysed articles, 6 used Spearman correlation to examine relationships between variables, 7 used Pearson correlation, and 1 applied both Spearman and Pearson correlations to ensure robustness and compare the strength and direction of correlations. These diverse approaches highlight the variety of statistical methods adopted for association analysis across different studies, reflecting considerations such as data distributions and sample characteristics. More advanced predictive models control for potential confounders such as population size, changes in testing practices over time, and environmental variables like temperature and wastewater flow rates [110,111]. These adjustments enhance prediction accuracy and serve as powerful tools for early detection and monitoring, enabling public health agencies to predict outbreaks.

Statistical models have been crucial not only in confirming the importance of viral RNA detection and validating wastewater surveillance methodologies but also in enhancing these predictive capabilities. They facilitate early prediction of infection surges, thereby improving the reliability and speed of public health responses. Recent studies increasingly employ complex statistical models that not only correlate environmental with clinical data but also identify correlations within different segments of time-series data. Agent-Based Modelling constructs models linking agents to estimate population immunity and infection dynamics, integrating wastewater data for a comprehensive public health perspective [112]. Joinpoint regression provides a robust framework for analysing wastewater data in relation to clinical outcomes, revealing insights that simpler statistical techniques might overlook [113].

**Table 4:** Summary of variables involved in SARS-CoV-2 WBE modelling.

No. Crt.	Estimated Lag Period (Days)	Modeling Technique	References
1.	0	Spearman correlation	[114]
2.	2	Pearson’s correlation	[111]
3.	22–24	Spearman correlation	[115]
4.	3	Spearman correlation	[116]
5.	4	Linear Regression model	[117]
6.	4–6	Generalised Additive Models (GAMs)	[118]
7.	3–9	Pearson, Spearman correlation	[119]
8.	5.5	Susceptible-Exposed-Infectious-Recovered (SEIR) model with Gamma distribution	[109]
9.	6	Poisson distribution	[120]
10.	6.2	Approximate Bayesian computation	[121]
11.	3	Cross correlation	[122]
12.	14	Spearman correlation	[123]
13.	14	Monte Carlo simulation	[63]
14.	14	Regression models Simple Linear, Double Square Root, Square Root-Y	[124]
15.	14–21	N.R.	[125]
16.	21	Pearson correlation	[126]
17.	19–21	N.R.	[127]
18.	28	Pearson correlation	[108]
19.	2–7	Pearson correlation	[107]
20.	4–7	Pearson correlation	[128]
21.	7–14	Spearman correlation	[129]
22.	5–9	Pearson correlation	[130]
23.	6–8	Linear Regression	[20]
24.	10	Poisson distribution	[131]

N.R.—Not reported.

SARS-CoV-2 monitoring in wastewater is a useful tool for epidemiological surveillance and may yield early insights into community infection trends. However, the lack of standardised protocols and the variables related to methodologies and the environment call for a strong correlation with clinical epidemiological data. These will strengthen the reliability of wastewater data and allow timely public health interventions and solutions to be developed [28,125]. The literature points to a statistically significant relationship between the number of COVID-19 clinical cases and the wastewater detection rates, along with concentration levels for SARS-CoV-2. Most studies reported rising viral RNA loads in wastewater with an increase in clinical cases, therefore offering a predictive tool for public health surveillance. However, several studies observed a contrast regarding the relationship between clinical cases and the reported concentration of SARS-CoV-2 in wastewater. The discrepancies of their results have been attributed to such factors as the inconsistencies in sampling and decay of viruses in wastewaters, as well as differences in sewage infrastructure [121,122]. These exceptions point towards the need for standardised protocols and further research on factors affecting WBE.

### 3.6. Standardisation Efforts and National Approaches in SARS-CoV-2 Wastewater Surveillance

Studies using surveys or data obtained from local authorities have attempted to describe the status and situation of wastewater monitoring in broad regions such as Europe and the United States of America (USA) [132,133]. In the USA, for example, the wastewater surveillance process has been supported by the National Wastewater Surveillance System (NWSS), which is meant to coordinate and receive data from state, territorial, and local health departments and take mitigation measures accordingly. The implementation of this system helped in guiding public health actions and unify the local wastewater surveillance strategies [56,133]. Similarly, Europe also launched a programme (EU Wastewater Observatory for Public Health) in order to gather wastewater surveillance information data from European countries and take action. Nevertheless, many countries across the globe implemented programmes to maintain good communication between laboratories and national governments. South Africa through “South Africa National Institute for Communicable Diseases”, Canada with the “Government of Canada COVID-19 wastewater monitoring dashboard”, Japan with “New Integrated Japanese Sewage Investigation for COVID-19” and India through “The Pune Wastewater Surveillance (WWS) project” are just a few examples of government-implemented systems and programmes for monitoring SARS-CoV-2 in wastewater [134]. One of the ways these programmes contributed to national and international surveillance is the standardisation of best practices in different laboratories [132]. Additionally, these programmes are meant to use the data to discover outbreaks before clinical surveillance and take measures in advance, such as population immunisation through vaccines, physical distancing, mask mandates, large-scale testing, etc. [135].

The efforts previously undertaken by governments to enhance wastewater surveillance across regions and countries not only supported efforts to mitigate the effects of the SARS-CoV-2 pandemic but also underscored the need to standardise and adopt similar workflows for other viruses, pathogens, and antibiotic-resistant bacteria. By developing dashboards and platforms for surveillance data, governments strengthened international frameworks that will further assist in monitoring other pathogens [136]. Additionally, there has been a shift in the correlation between clinical cases and wastewater surveillance, attributable to evolving testing practices for the novel coronavirus. This change is primarily due to the increased use of at-home test kits. These results highlight the importance of wastewater surveillance programmes in regions where clinical testing is underreported [137].

Given that many techniques have been used in wastewater monitoring, numerous studies on the detection of SARS-CoV-2 in wastewater have highlighted the importance of standardisation. This is essential to ensure the accuracy and reliability of results and to allow comparisons between different public health organisations [132,138]. Oyervides-Muñoz [139] evaluated the reproducibility and reliability of the qRT-PCR methodology across five laboratories in Mexico City through statistical analysis. The research aimed to demonstrate that consistent reproducibility and stability of standardised methods across all collaborators are vital for accurate SARS-CoV-2 detection in wastewater monitoring systems. The measurement results ranged from 251.46 to 54,548.84 copies/L, aligning with the expected calibration curve range and demonstrating valid, precise readings. The variations in viral load measurement between laboratories were attributed to inconsistent sample recovery rates before treatment, which affected reproducibility [139]. Two EU surveys were conducted in over 750 WWS laboratories to assess activities related to wastewater monitoring, alongside the methods and quality control for SARS-CoV-2 monitoring. The surveys showed that laboratories deployed rigorous strategies for viral detection, but results varied due to a lack of standardisation. Improved communication between laboratories and the establishment of certified materials are among the measures that should be adopted to further harmonise wastewater testing [132].

WBE has been used as a tool for monitoring not only SARS-CoV-2 but also other viruses found in treated and untreated wastewater. This type of surveillance has been applied to the monitoring of various pathogens, including enteroviruses, noroviruses, monkeypox viruses, polyoviruses, and influenza viruses. This method was used, for example, to assess the circulation of the poliovirus in the population and the efficiency of immunisation [140,141]. Monitoring efforts regarding influenza started after the potential of WBE epidemiology was explored further during the COVID-19 pandemic [142]. As influenza has a high probability of causing pandemics, wastewater surveillance can be regarded as a valuable tool for predicting flu epidemics [143]. Similar studies have been conducted on the norovirus to determine its prevalence in communities, showing the importance of WBE in regions with limited clinical surveillance [144,145]. To further benefit from this early warning system, future perspectives should focus on integrating it into clinical monitoring. An integrated system could provide information about ongoing outbreaks, enabling early intervention [146]. The approach was adopted at the University of Denver’s campus, where wastewater was monitored for SARS-CoV-2, and individuals were screened using high-sensitivity testing. This allowed individuals from dormitories with confirmed SARS-CoV-2 wastewater samples to be prioritised for testing. The results showed that combining the two testing methods can efficiently mitigate the spread of the virus in the community [147]. This integrated approach shows promise in interrupting outbreaks and mitigating the spread of viruses in communal living facilities.

The COVID-19 pandemic has had a profound impact on medical infrastructure, social life, and the economy. Despite the development of various vaccines to prevent the spread of the virus, mutations resistant to existing vaccines have been identified. As a result, the virus continued to spread even after the pandemic concluded [148]. As observed by Tang [149], the emergence of new SARS-CoV-2 strains, such as the latest Omicron subvariants, raises concerns about the potential for accelerated transmission through new animal reservoirs [149]. Therefore, wastewater epidemiology plays a vital role in monitoring pathogens in sewage systems, aiding in public health improvements and preparing for future pandemics [150].

## 4. Limitations

This systematic review was conducted according to the PRISMA guidelines and used publications from the WoS and PubMed databases. Although using two major databases and a clearly defined time span (January 2020–October 2024) strengthens the study’s methodology, limiting the search to English-language, peer-reviewed articles may have introduced language bias, resulting in the exclusion of relevant studies published in other languages. Additionally, excluding gray literature such as preprints, government or technical reports, and non-peer-reviewed studies may have affected the completeness of the evidence base, especially in a rapidly evolving research field like SARS-CoV-2 wastewater surveillance. The use of specific keywords may have resulted in the omission of studies that addressed similar topics but employed different terminology. Focusing the inclusion criteria exclusively on the surveillance and quantification of SARS-CoV-2 in wastewater limited the scope of the review, thereby excluding research related to the broader environmental or clinical aspects of the disease. Another limitation is the heterogeneity of the selected studies, which hindered direct quantitative comparisons and necessitated predominantly qualitative interpretations of the findings. To preserve data accuracy and respect the original reporting of the cited studies, the original units of measurement were retained. Despite these limitations, this review’s systematic approach provides a comprehensive and balanced overview of the current scientific evidence on SARS-CoV-2 surveillance in wastewater, offering a solid foundation for future research in this area.

## 5. Conclusions

In this systematic review, we analysed research efforts through a critical and reproducible screening of reviewed articles from January 2020 to October 2024 on analytical methods for surveillance of SARS-CoV-2 and its variants in wastewater. The relevant data were retrieved from the two databases, PubMed and WoS. In particular, this analysis brings together a variety of methods of analysis. The most commonly used method to determine SARS-CoV-2 viral load was qRT-PCR. Regarding NGS technology, a variety of sequencing platforms, such as Illumina and Oxford Nanopore, have been successfully used to monitor circulating variants before clinical cases were reported. By exploring more of the genetic diversity of SARS-CoV-2, studies identified the variants present in different communities and observed how public events influenced the introduction of variants within regions. The geographical distribution showed that monitoring SARS-CoV-2 has been a global effort. Furthermore, our findings indicate that the majority of studies have identified a correlation between trends in wastewater SARS-CoV-2 RNA concentrations and epidemiological data. In order to maximise the impact of WBE in public health, it is essential that both governments and the public sector actively integrate wastewater surveillance into routine health monitoring programs. This integration enables early detection of outbreaks, evidence-based policy decisions, and resource allocation for targeted interventions.

As a future perspective for WBE to be seamlessly incorporated into public health strategies, it is imperative to standardise methodologies to ensure the collection of comparable and reliable data. The establishment of globally recognised protocols for sample collection, data processing, and interpretation will enhance the accuracy of routine health monitoring, thereby facilitating more proactive and evidence-based public health responses. In addition to the application of these methodologies to SARS-CoV-2, there has been an increasing use of these methods with other pathogens, including influenza and antimicrobial resistance genes. This underscores the broader significance of WBE in the context of infectious disease surveillance and the importance of public health preparedness in taking quick actions against potential future epidemics.

## Figures and Tables

**Table 1:** Search method.

Criteria	Query	No. of Results in WoS	No. of Results in PubMed
1	(“waste water” OR “wastewater” OR “wastewater treatment plant” OR “WWTP” OR “untreated wastewater” OR “sewerage system” OR “sewage” OR “river” OR “untreated wastewater”) NOT (review)	261,384	84,167
2	(“Human Coronavirus” OR “SARS Virus” OR “Severe acute respiratory syndrome” OR “SARS-CoV-2” OR “COVID-19” OR “COVID19” OR “2019-nCoV” OR “SARS-CoV” OR “severe acute respiratory syndrome coronavirus 2” OR “HCoV” OR “nCoV” OR “Novel coronavirus 2019” OR “2019 novel coronavirus” OR “Wuhan coronavirus” OR “novel coronavirus” OR “coronavirus 2019” OR “novel coronavirus disease”) NOT (review)	393,065	370,210
3	(“detection” OR “quantification” OR “RT-PCR” OR “qRT-PCR” OR “dPCR” OR “PCR” OR “Polymerase Chain Reaction”) NOT (review)	881,717	511,099
4	(“WBE” OR “monitoring” OR “surveillance” OR “monitoring system” OR “surveillance system” OR “wastewater-based epidemiology” OR “wastewater-based epidemiology surveillance” OR “wastewater-based epidemiology” OR “environmental monitoring” OR “wastewater-based”) NOT (review)	474,040	318,545
5	(“RNA” OR “ribonucleic acid” OR “nucleic acid” OR “viable particles”) NOT (review)	243,758	277,156
	1 AND 2 AND 3 AND 4 AND 5(“waste water” OR “wastewater” OR “wastewater treatment plant” OR “WWTP” OR “untreated wastewater” OR “sewerage system” OR “sewage” OR “river” OR “untreated wastewater”) AND (“Human Coronavirus” OR “SARS Virus” OR “Severe acute respiratory syndrome” OR “SARS-CoV-2” OR “COVID-19” OR “COVID19” OR “2019-nCoV” OR “SARS-CoV” OR “severe acute respiratory syndrome coronavirus 2” OR “HCoV” OR “nCoV” OR “Novel coronavirus 2019” OR “2019 novel coronavirus” OR “Wuhan coronavirus” OR “novel coronavirus” OR “coronavirus 2019” OR “novel coronavirus disease”) AND (“detection” OR “quantification” OR “RT-PCR” OR “qRT-PCR” OR “dPCR” OR “PCR” OR “Polymerase Chain Reaction”) AND (“WBE” OR “monitoring” OR “surveillance” OR “monitoring system” OR “surveillance system” OR “wastewater-based epidemiology” OR “wastewater-based epidemiology surveillance” OR “wastewater-based epidemiology” OR “environmental monitoring” OR “wastewater-based”) AND (“RNA” OR “ribonucleic acid” OR “nucleic acid” OR “viable particles”) NOT (review)	548	509

**Table 2:** Decision-making framework for the methodological implementation of wastewater-based epidemiology (WBE) programs.

Implementation Level	Objective	Matrix Type	Sampling Method	Extraction Strategy	Detection & Analysis
BASIC	Early warning	Raw influent	Grab or composite	Standard commercial viral RNA kits	qRT-PCR
STANDARD	Trend monitoring	Primary sludge (or influent)	24 h composite	Robust kits with enhanced inhibitor removal	dPCR/ddPCR (Superior for high-inhibition matrices)
ADVANCED	Genomic surveillance	Raw influent	24 h composite	High-purity extraction	NGS (Illumina/Nanopore) and bioinformatics

**Table 3:** A comparison of the most effective detection methods in the field of WBE.

Method	Sensitivity	Feasibility	Specificity	Advantages	Disadvantages
qRT-PCR	High	High Extensive accessibility	High	This product is widely used, delivering rapid results with quantifiable outcomes	Affected by PCR inhibitors
dPCR	Very high	Moderate Specialised equipment is necessary for this process	High	It offers higher precision and absolute quantification	Higher cost. The processing time is longer, and the workflow is complex
ddPCR	Very high	Moderate: This process requires specialised equipment	High	It offers higher precision and absolute quantification	Higher cost The processing time is longer, and the workflow is complex
Sanger Sequencing	Moderate	Low	High	This technology is accurate for specific genes and cost-effective for small-scale studies	This system has two main limitations: low throughput and limited ability to detect multiple variants simultaneously. As a result, it is not suitable for variant discovery
Illumina Sequencing	High	Low This field requires a high level of expertise in bioinformatics	High	The system is characterised by its high throughput capacity and its ability to detect variants with a high degree of accuracy	The process is both costly and time-consuming due to the necessity of complex library preparation techniques. Furthermore, the utilisation of short reads imposes limitations on the comprehensive assembly of genomes
Oxford Nanopore Sequencing	High	Moderate Portable alternatives	High	This technology is characterised by its mobility, the capacity for real-time sequencing, and the capability to generate long reads	The implementation of bioinformatics expertise is essential, and the necessity for error correction has been identified

## Data Availability

All the data were obtained from publicly available information.

## Supplementary Materials

The following supporting information can be downloaded at https://www.mdpi.com/article/10.3390/v18020205/s1, References [151,152,153,154,155,156,157,158,159,160,161,162,163,164,165,166,167,168,169,170,171,172,173,174,175,176,177,178,179,180,181,182,183,184,185,186,187,188,189,190,191,192,193,194,195,196,197,198,199,200,201,202,203,204,205,206,207,208,209,210,211,212,213,214,215,216,217,218,219,220,221,222,223,224,225,226,227,228,229,230,231,232,233,234,235,236,237,238,239,240,241,242,243,244,245,246,247,248,249,250,251,252,253,254,255,256,257,258,259,260,261,262,263,264,265,266,267,268,269,270,271,272,273,274,275,276,277,278,279,280,281,282,283,284,285,286,287,288,289,290,291,292,293,294,295,296,297,298,299,300,301,302,303,304,305,306,307,308,309,310,311,312,313,314,315,316,317,318,319] are cited in the Supplementary Materials. Table S1. A description of the analytical methods employed in selected literature.
