# Supplementary material for: A Systematic Review of Methodological Approaches to SARS-CoV-2 Wastewater Surveillance
Source: Viruses. 2026 Feb 4;18(2):205. doi: 10.3390/v18020205 (PMC12945004; doi:10.3390/v18020205)
Supplement: Supplementary file 1 [file viruses-18-00205-s001.zip › viruses-4102633-supplementary.pdf]

viruses MDPI

Supplementary Table S1. A description of the analytical methods employed in selected literature.

Surveillance Period	Wastewater type/sample	Zone/location	Concentration method	Detection and sequencing methods	Results (Concentration PCR/variants)	References
03 February 2020–02 April 2020	Wastewater treatment plants	Italy: Milan, Rome	Two-phase (PEG dextran method) separation	Nested RT–PCR, qRT-PCR /ORF1ab, S, RdRP	50% positive rate, not available.	[151]
23 April 2020–5 May 2020	Wastewater pumping stations, plant inlets and outlets. wastewater treatment plants	Italy: Paduna, Veneto Region	Ultrafiltration	qRT-PCR assay/ORF1b, N gene	The LOD was 2.5 G.C. per a minimum SARS-CoV-2 titre of 4.8–4.9 Log_{10} G.C./L.	[152]
October 2021-April 2022	Wastewater Treatment Plants	Italy: Campania	PEG precipitation	qRT-PCR / RdRP, E (Corman et al. 2020)	155 out of 168 samples (92.3%) tested positive for SARS-CoV-2 with concentrations ranging from 1.73 × 10^2 G.C./L to 2.90 × 10^5 G.C./L (Napoli OVEST—North) and 2.11 × 10^4 (Napoli EST) G.C./day*inhabitant to 5.76 × 10^7 G.C./ day*inhabitant (Area Casertana)	[45]
October 2021–December 2022	Wastewater Treatment Plants	Italy: Sicilia, Liguria, Lombardia, Emilia Romagna	PEG precipitation	Amplification: qRT-PCR / ORF1ab (La Rosa et al. 2021) Sequencing: Sanger/NGS through Istituto Superiore di Sanità - Rome, Italy	Amplification: Positivity rate of 94.6% (985/1041); The median viral load was 6350 (G.C./L) (range 28–975,000) Sequencing: Delta (B.1.617.2), Omicron (B.1.1.159, BA.1, BA.2, BA.4, BA.5, XBB.1)	[153]
30 March 2020–15 June 2020	Wastewater treatment plants	Italy: Lombardy	PEG/NaCl centrifugation	RT–PCR, ddPCR/N1, N3 genes (CDC, US)	A 61% positivity rate. The highest concentration was detected in Brembate (2.10 + 05 copies/L).	[154]
May 2020–November 2020	Wastewater treatment plants	Italy: Bari, Barletta-Andria-Trani, Brindisi, Foggia, Lecce, Taranto	Two-phase separation (the PEG–dextran method)	qRT-PCR/ ORF1ab (La Rosa, 2021)	SARS-CoV-2 positivity rate was 12.4% (26/210) with concentrations ranging between 8.8 × 10^2 to 6.5 × 10^4 G.C./L	[155]
October 2021–September 2022	Wastewater treatment plants	Italy: Tuscany	PEG precipitation	qRT-PCR/ ORF1ab (La Rosa et al. 2021)	In 2021, viral load decreased from winter (6.4 ± 2.3 Log_{10} G.C./100,000 inh/day) to spring (5.7 ± 2.1 Log_{10} G.C./100,000 inh/day) and summer (5.3 ± 0.9 Log_{10} G.C./100,000 inh/day). In 2022 viral loads were consistent during winter, spring and summer: 6.8 ± 2.2 Log_{10} G.C./100,000 inh/day, 6.7 ± 2.1 Log_{10} G.C./100,000 inh/day, and 6.0 ± 2.1 Log_{10} G.C./100,000 inh/day	[156]
March–October 2021	Wastewater treatment plant	Italy: Florence	PEG precipitation	qRT-PCR, ddPCR/ ORF1b (La Rosa et al. 2021) N, E (Promega), N1, N2 (COVID-19 kit CE-IVD)	Positivity rate varied among methods: 8/35 (22.9 %) (Method A), 14/35 (40.0 %) (Method B), 19/35 (54.3 %) (Method C) and 19/35 (54.3 %) (Method D); Concentration values among all methods were	[157]

					from 3.8×10^2 and 2.2×10^5 G.C./L	
September 2020–February 2021	Wastewater treatment plant, flow discharge	Italy: Venice	Ultrafiltration	Amplification:qRT-PCR/ E_Sarbeco (Corman et al. 2020) N1, N2 (CDC, US) Sequencing: Illumina Miseq/ ARTIC V3	Amplification: 41 (45.6%) of samples were positive for SARS-CoV-2. The highest detection rate was found in sludge with mean values of 2.64×10^6 G.C./L for Civil Hospital (CH) and 2.02×10^6 G.C./L for Sacca Fisola (SF). Sequencing: B.1.177 and B.1.160 variants were detected	[158]
09 July 2020–07 April 2021	Wastewater treatment plants	Brazil: ABC Region, São Paulo	PEG precipitation	qRT-PCR /N1, N2 genes (CDC US)	A total of 53% (116/220) were positive for N1 and 34% (74/220) were positive for N2 gene assays; maximum concentrations of 7.2 for N1 and 7.7 for N2 Log_{10} G.C. L^{-1}.	[63]
April–August 2020	Wastewater treatment plants, sewer pipes, slums	Brazil: Niterói	Ultracentrifugation	Amplification: qRT-PCR /N2 (CDC, US) Sequencing: Illumina MiSeq	Amplification: 84.3% (188/223) positivity rate; concentration ranged between 3.1 to 7.1 log_{10} G.C./100 mL Sequencing: B.1.1.33 (clade G) was detected	[159]
15 August 2021–19 September 2021	Household waste (leachate)	Brazil: City of São Paulo	PEG precipitation	qRT-PCR /N1, N2 (CDC, US)	A 75% positive rate for N1 and 42% for N2; mean concentrations: 4.2 for N1 and 4.0 (L^{-1} for N2 Log_{10} G.C.).	[160]
15 July 2020–15 July 2021	Raw wastewater	Brazil, São José do Rio Preto	Centrifugation	qRT-PCR /N1, N2 (CDC, US)	A 100% positive rate for N1 and 96.6% for N2; concentrations ranged from 1×10^3 copies/L to 1.3×10^5 copies/L for the N1 target in raw sewage and from 0 to 8.6×10^4 copies/L for the N2 target.	[65]
May 2020–May 2021	Wastewater treatment plants	Brazil: Porto Alegre	Ultracentrifugation	qRT-PCR / N1, N2 (CDC, US)	Viral loads ranged between 1×10^6–3×10^6 G.C./L.	[161]
July 2021–July 2022	Wastewater treatment plants	Brazil: Lavras, Southern Minas Gerais State	Electronegative membrane	qRT-PCR / N1 (CDC, US)	Concentration values: minimum of 221.0 and maximum of 2.359 (copy numbers/L), mean of 890.7 (G.C./L)	[162]
January–August 2021	Wastewater treatment plant	Brazil: Goiânia, Goiás	PEG precipitation	qRT-PCR / N1, N2 (IDT)	43.63% (24/55) positivity rate; N1 had a mean of 3.23 Log_{10} G.C./L (ranging from 2.73 to 3.73 Log_{10}) and N2 had a mean of 2.95 Log_{10} G.C./L (ranging from 2.69 to 5.47 Log_{10})	[52]
16 September 2020–08 August 2021	Wastewater treatment plant, public water fountains, stream	Brazil: Porto Alegre, Novo Hamburgo and São Leopoldo	Ultracentrifugation	qRT-PCR /N1, N2 (CDC, US), E (Corman et al. 2020)	Stream SARS-CoV-2 concentration: 4.65×10^3 G.C./L– 5.19×10^6 G.C./L Fountain SARS-CoV-2 concentration: 2.41-8.59–10^1 G.C./L Sewage SARS-CoV-2 concentration: 5.42×10^4 G.C./L–2.54×10^6 G.C./L	[163]
3 January, 2021–22 January 2022	Wastewater treatment plants, lift stations, sewer manholes	Brazil: ABC Region, Foz do Iguaçu, Goiânia, and Federal District	PEG precipitation method	qRT-PCR / N1, N2 (CDC, US)	Highest viral loads ranged between 10^6–10^8 G.C./L	[164]
14 April 2021 – 4 August, 2021	Wastewater treatment plant, sewer manhole	Brazil: ABC Region, São Paulo	PEG precipitation	qRT-PCR/ N1, N2 (CDC, US)	Composite samples had a positivity rate of 88.2% (15/17) and 76.5% (13/17) for N1 and N2 genes. The mean concentration was	[165]

https://doi.org/10.3390/v18020205

					5.3 log_{10} G.C./ L^{-1} for N1 and 6.1 log_{10} G.C./ L^{-1} for N2 Grab samples had a positivity rate of 75.0% (12/16) for both genes. The mean concentration was 5.5 log_{10} G.C./ L^{-1} for N1 and 6.4 log_{10} G.C./ L^{-1} for N2	
November 2021–October 2023	Wastewater treatment plant	China: Shijiazhuang City, Hebei province	Membrane filtration (nitrocellulose)	RT-ddPCR /N1 (CDC, US), E (Charité-E) genes	The concentration of the N1 gene in 62 positive samples ranged from 625 to 23,900 copies/mL, and the concentration of the E gene ranged from 228 to 17,800 copies/mL.	[64]
10 August 2022–09 September 2022	Wastewater treatment plant	China: Longhua	Adsorption-precipitation	qRT-PCR /N, ORF1ab (Wuhan EasyDiagnosis Biomedicine Co., Ltd)	Samples having a concentration below 200 copies/mL had a recovery rate between 10-60% for N gene and 1.6-28% for ORF1ab	[166]
27 December 2022–6 January 2023	Sewage manholes	China: Chengdu	PEG precipitation	Amplification: qRT-PCR / ORF1ab, N (CCDC, China) Sequencing: ATO-Plex V3.1/ Primer Pool 1 V3.1 and PCR Primer Pool 2 V3.1	Amplification: Concentration of SARS-CoV-2 RNA was 10^4–10^5 G.C./L Sequencing: BA.5.2, BA.5.2.49, BA.5.2.48, BF.7.14, BF.7.14.4 DY.1, DY.2, DY.3 and DZ.1 sublineages were detected	[167]
June 8–September 29, 2020	Sewage Treatment Work (STW), Sewage Screening Plant (SSP), and Sewage Pumping Station (SPS), manholes	Hong Kong	Ultrafiltration	qRT-PCR/ N (Integrated DNA Technologies)	21% (23/107) positivity rate for SARS-CoV-2; 1975 copy/mL was the highest recorded concentration; Among the large sewage facilities (STW, SSP, and SPS), 12/87 samples were positive, with concentrations ranging from 0.3 to 177 copies/mL.	[168]
June 2020–August 2021	Wastewater influent	Ireland: Dublin	Ultrafiltration	Amplification: qRT-PCR, RT-ddPCR /N1 (CDC, US), N501Y (Alpha variant), L452R (Delta variant) (Bio-Rad) Sequencing: GridION platform/Q5 Hot Start High-Fidelity DNA polymerase, Luna script (NEB)	Amplification: The L452R variant had the highest concentration in May and June 2021, N501Y had the highest concentration on the 6th of January 2021 Sequencing: lineages B.1.177, B.1.258, and AZ, including VOCs for the Alpha and Delta variants.	[114]
October 2020–February 2021	Wastewater treatment plant	Ireland: Dublin	Ultrafiltration	qRT-PCR/ N1 (CDC, US)	The concentration of SARS-CoV-2 at the Ringsend WWTP ranged from 1450 to 12100 G.C./100 mL (median=4355). At the Shanganagh WWTP, the concentration ranged from 552 to 8540 G.C./100 mL (median=1890).	[169]
March–July 2020	Untreated influent and treated effluent wastewater	Wales and Northwest England, UK: Gwynedd, Cardiff, Liverpool, Manchester, the Wirral and Wrexham	Ultrafiltration	Amplification: qRT-PCR /N1 (CDC, US), E (Sarbeco) Sequencing: Illumina MiSeq, V3 nCov-2019 primers (ARTIC)	Amplification: concentrations ranged from $<1.2\times10^3$ (<LOQ) to the highest recorded concentration of 1.5×10^4 G.C. 100 mL^{-1} Sequencing: 25 and 75% of the SARS-CoV-2 genome was recovered; 702 unique SNP sites and 267 indels were detectable across the 84 samples.	[70]
25 October 2021–26 January 2022	Campus sewage	Wales: Bangor	PEG precipitation	Amplification: qRT-PCR / N1 (CDC, US)	Amplification: the highest concentrations were 1.2×10^4 G.C./sampler (Site	[170]

				Sequencing: Illumina NextSeq 1000/ P1 kit	4), $1.1x10^4$ G.C. /sampler (Site 3), 7.67 $x10^3$ G.C. /sampler (Site 2); the lowest SARS-CoV-2 concentration was $5.8x10^3$ G.C./sampler (Site 1). Sequencing: Delta and Omicron variants were detected	
25 September 2020–22 March 2021	Wastewater treatments plants	Wales: Chester, Wrexham, Flint, Kinmel Bay, Conwy, Bangor, Llangefni and Holyhead	PEG precipitation	qRT-PCR / N1 (CDC, US)	The concentration varied from 68 to 1,420,863.5 G/L across the locations. The mean viral load for each site was as follows: Bangor: 11,012 G.C./L; Chester: 270,859 G.C./L; Conwy: 25,544; G.C./L; Flint: 19,202 G.L./L; Holyhead: 10,093 G.C./L; Kinmel Bay: 17,284 G.C./L; Llangefni: 8,722 G.C./L; Wrexham: 18,948 G.C./L	[93]
May 2020–February 2022	Sewage influent	Scotland	Centrifugation with filter	qRT-PCR /N1 gene	A total of 9.3% of the analysed samples were "negative", 7.3% were "weak positive", 14% were "positive (DNQ)", and 63.4% were "positive". The LOD was set to 1,316 G.C./L and the LOQ was 11,368 G.C./L.	[171]
October 2020–July 2021	School wastewater	England	PEG precipitation	Amplification: qRT-PCR/N1, E (Sarbeco) Sequencing: Illumina NovaSeq 6000, EasySeq™ RC-PCR SARS CoV-2 Whole Genome Sequencing kit v3.0 (NimaGen)	Amplification: N1 and/or E were detected in 31.8% of samples (272). N1 was detected in more samples than the E gene and 33.1% had both genes present. The number of gene copies detected ranged from $1.3x10^3$ G.C./L (LOD) to $9.2x10^6$ G.C./L for the N1 gene and from $3.0x10^3$ G.C./L (LOD) to $1.3x10^6$ G.C./L for the E gene. Sequencing: Alpha (B.1.1.7) last confirmed detection on 10th February 2021; Delta (B.1.617.2) first confirmed detection on 08 June 2021. Delta's sublineages AY.43, AY.46.5, and AY.2 were also detected.	[71]
2 April–28 May 2020	Wastewater treatment plant	France: French Grand Est region	Ultrafiltration/PEG precipitation	qRT–PCR, RT-digital droplet PCR (RT-ddPCR)/E (Sarbeco)	The mean concentrations reached $2.1×10^7 ± 1.1×10^7$ G.C./L and $1.6×10^7 ±$ $1.4×10^7$ G.C./L in unconcentrated and concentrated samples, respectively. The LOQ for the E gene reached $2×10^2$ G.C./L.	[172]
14 April–9 June 2021	Wastewater treatment plants	France: Paris	Ultrafiltration	dPCR/ N1 (PENTA-CoV kit)	Average concentration values for the three WWTPs were: 236 GU/mL for SEV, 273 GU/mL for SEG, and 481 GU/mL for SEM	[173]
5 March–23 April 2020	Wastewater treatment plants	France: Paris	Ultracentrifugation	qRT-PCR / E, RdRp	The highest concentration of SARS-CoV-2 was $2.5×10^6$ U.G./L	[174]
February–October 2022	Wastewater treatment plants	France: Alsace region and French Guiana	Ultrafiltration	Amplification: qRT-PCR/ E_Sarbeco (Corman et al. 2020), RdRp Sequencing: Illumina NovaSeq 6000/Artic v4.1	Amplification: Viral load ranged from $5.9×10^1$ to $5.5×10^3$ and from $3.8×10^3$ to $2.5×10^5$ G.C./L Sequencing:	[175]

					Optimal sequencing concentration of SARS-CoV-2 RNA in the samples was 2.0×10^5 G.C./L	
January–October 2023	Wastewater treatment plant	France: Grand Est, Nouvelle Aquitaine, Provence Côte d'Azur, Normandie, French Guiana	Ultrafiltration	Amplification: qRT-PCR, RT-ddPCR/ E (Corman et al. 2020) Sequencing: Illumina NovaSeq 6000 /ARTIC v4.1	Successful sequencing was achieved by samples below 5×10^4 G.C./L, that were concentrated by ultrafiltration; samples with viral load above 5×10^4 G.C./L did not need ultrafiltration for sequencing	[176]
December 2020–February 2022	Municipal catchment	Austria	PEG precipitation	Amplification: qRT-PCR, RT-dPCR/ N (CDC, US) Sequencing: NovaSeq 6000 platform (Illumina), ARTIC v3	Sequencing: Alpha (B.1.1.7), Delta (B.1.617.2), Omicron (BA.1 and BA.2), B.1.160 and B.1.258	[74]
4 October 2020–5 May 2021	Sewer system	Austria: Vienna	PEG precipitation	Amplification: qRT-PCR / N (ViroReal® Kit SARS-CoV-2 & SARS) Sequencing: Illumina NovaSeq 6000/ ARTIC V3 primers	Amplification: Viral load in wastewater reported to PE11 (population equivalent) ranged from 5.01×10^7 to 1.10×10^9 G.C./PE_{11}, as per active case it ranged between 1×10^{11} to 3.5×10^{11} G.C./PE_{11} Sequencing: Alpha lineage (B.1, B.1.1, B.1.1.7, B.1.160, B.1.177, P.1.12, Q.4)	[177]
January–February 2021	Wastewater treatment plant	Luxembourg: Luxembourg City	Ultrafiltration	qRT-PCR /N1, N2, N3 (CDC, US) E (Sarbeco), ORF1a (La Rosa, 2021), and RdRP (Corman et al. 2020) genes	Concentrations in the raw sewage sample averaged $1.0 \pm 0.6\times10^4$ G.C./L and $3.8\pm0.1\times10^2$ G.C./ L.	[58]
November 2020–March 2021	Wastewater treatment plants	Luxembourg: Beggen (BEG), Bettembourg (BET), Bleesbruck (BLEE), Boevange (BOEV), Echternach (ECH), Grevenmacher (GRE), Hespérange (HESP), Mersch (MER), Pétange (PET), Schifflange (SCH), Troisvierges (VIE), Uebersyren (UEB) and Wiltz (WIL)	Ultrafiltration	Amplification:qRT-PCR/ E, RdRP, N, S (Allplex™ SARS-CoV-2 Assay kit) Sequencing: Illumina MiniSeq or Miseq/ ARTIC V1	Sequencing: B.1.221, B.1.160, or B.1.177, B.1.351, B.1.1.7, B.1.258, B.1.1.420, B.1.474 were the main detected lineages	[57]
July 1, 2021–December 31, 2021	Wastewater treatment plants	Switzerland: Basel	Maxwell® RSC Environ Waste-water TNA Kit (Promega, Madison, USA	Amplification: qRT-PCR/N1, N2, E genes, Sequencing: NGS/ARTICv4 protocol	Amplification: The first peak was in August 19, 2021 with values of 1.99 $\times10^5$ G.C./L wastewater. The highest levels were measured on December 7, 2021, with a SARS-CoV-2 RNA titre of 4.13×10^5 G.C./L wastewater and 1.08×10^{13} G.C./100000 inhabitants. Sequencing: The B.1.617.2 Delta variant of SARS-CoV-2 was predominant before December 29, 2021; from November 21, 2021, mutations associated with the B.1.1.529 variant were traceable.	[123]
September 2020–March 2021	Influent wastewater	Liechtenstein	PEG precipitation	Amplification: qRT-PCR/ N1 (CDC US) Sequencing: Illumina NovaSeq 6000/ Arctic V3 Protocol	Sequencing: B.1.1.7 variant was detected.	[117]

October–December 2020	Wastewater Treatment Plant	Turkey: Çorum	Sorbent method	qRT-PCR / N, ORF1ab (SARS-CoV-2 Double Gene RT-qPCR kit (Bio-Speedy)	42.8% (3/7) of samples were positive for SARS-CoV-2	[178]
February 5–March 25 2020	Wastewater treatment plants	The Netherlands: Amsterdam Den Haag Utrecht Apeldoorn Amersfoort Schiphol Tilburg	Ultrafiltration	qRT-PCR /N1, N2, N3 (CDC, US), E (Sarbeco)	Concentrations of the N1 gene ranged from to 1.2×10^1 and 7.9×10^2G.C./mL, of the N2 gene ranged from 1.2×10^1 to 2.2×10^3 G.C./mL) and of the N3 gene ranged between $1.2x10^1$ and $1.8x10^3$ G.C./mL.	[13]
7 July 2020–1 December 2020	Wastewater treatment plants	Netherlands: Delf	Filtration on polyethersulfone, retention on a 1-mL diethylaminoethyl cellulose, electronegative membranes	qRT-PCR /ORF1ab, S, N (TaqPath COVID-19 RT-PCR Kit)	500 mL filtered samples average: S gene: $5.6x10^4$±$2.8x10^4$ viral gene copies L^{-1}; N gene: $6.0x10^4$±$2.6x10^3$ viral gene copies L^{-1}; ORF1ab gene: $7.5x10^4$ ± $2.4x10^4$ viral gene copies L^{-1} 50 mL filtered samples average: S gene: $1.3x10^4$±$1.0x10^4$ viral G.C./L^{-1}; N gene: $4.2x10^3$±$2.3x10^3$ viral G.C./L^{-1}; ORF1ab gene: $6.9x10^3$±$2.9x10^3$ viral G.C./L^{-1} 100 mL filtered samples average: S gene: $5.2x10^4$±$1.6x10^4$ viral gene copies L^{-1}; N gene: $9.7x10^3$ ± $6.7x10^3$ viral G.C./L^{-1}; ORF1ab gene: $2.9x10^4$±$4.7x10^3$ viral G.C./L^{-1}	[179]
26 March 2020–01 April 2020	Suburban pumping station	Australia: Brisbane	Electronegative membrane and ultrafiltration	Amplification: qRT-PCR/N (Sarbeco) Sequencing: Sanger and MiSeq Illumina/v3 MiSeq kit	The positivity rate was 2/9 (22%) with concentrations ranging between 1.9 and 12 G.C./100 mL.	[22]
17 December 2020–27 March 2021	Aircraft wastewater	Australia	Concentrator instrument (Concentrating Pipette Select™, CP Select™, InnovaPrep, Drexel, MO)	qRT-PCR/ N1, N2 (US CDC), N (CDC China)	SARS-CoV-2 was detected in 24 samples (64.9% of 37); N1 – mean of 3.17±0.90 Log_{10} G.C./50 mL wastewater, N2 (CDC US) – mean of 3.56±0.97 Log_{10} G.C./50 mL, N (CDC China) – mean of 3.22±0.81 Log_{10} G.C./50 mL	[180]
24 February–1 May 2020	Wastewater treatment plant	Australia: Brisbane	Electronegative membrane	Amplification: qRT-PCR/ N1, N2 (CDC), E_Sarbeco (Corman et al. 2020) Sequencing: Miseq Illumina/ N1 amplicons	Amplification: 21/63 samples were positive for SARS-CoV-2 with a viral load ranging from 135 to 11,992 G.C./100 mL of wastewater Sequencing: CDC N1 amplicons were mapped to their corresponding positions (i.e., 28,287-28,358) in the SARS-CoV-2 genome	[125]
August 2020	Wastewater treatment plant	Australia	Electronegative membrane	qRT-PCR/ N1 (CDC, US), N set (CCDC, China), N Sarbeco, E Sarbeco (Charité, Germany)	The positive ratio of each gene was 9/15 (60%), 8/15 (53.3%), 6/15 (40%) and 4/15 (26.7) of CDC-N1, CCDC-N, N-Sarbeco and E-Sarbeco, respectively	[181]
September 28, 2020–February 11, 2021	Wastewater Treatment Facilities (WWTF)	USA: Lebanon, Hanover, Woodsville, Nashua, and Concord, Hartford and Burlington	PEG precipitation	qRT-PCR and RT-ddPCR/N1, N2 (CDC, US)	Viral RNA positivity rate was 61.8% (175/283) by using qRT-PCR and 68.9%	[53]

					(195/283) by using RT-ddPCR	
March 2020–May 2020	Wastewater treatment plant, treated effluent discharge, untreated surface water, drinking water	U.S.A: Southern Nevada	Hollow fibre ultrafiltration, PEG precipitation, ultrafiltration	qRT-PCR/N1, N2 (CDC, US), E (Sarbeco), ORF1a (Lu et al. 2020) genes	At Facilities 1 and 2, respectively, N1 was detected in 64% and 9% of all samples, N2 was detected in 48% and 45%, E_Sarbeco was detected in 20% and 0%, and ORF1a was detected in 8% and 0% of samples. Concentrations at both facilities appeared to fluctuate between 10^4 and 10^6 G.C./L.	[182]
14 September 2020–21 September 2021	Downstream of dispense valves, manholes	U.S.A.: Tennessee	Ultrafiltration	qRT-PCR/ N1	Samples collected at different time intervals had the following SARS-CoV-2 concentrations: 3.60 ± 4.24 (1h), 2.77 ± 3.26 (2h), 3.06 ± 3.54 (4h), 2.83 ± 3.08 (6h), 3.22 ± 3.45 (24h) Log_{10} copies/L	[183]
December 20, 2020–November 16, 2021	Sewage collection systems (SCS)	U.S.A: Maryland	Ultrafiltration	qRT-PCR/N (PerkinElmer, Waltham, MA, USA)	The highest concentration was registered on February 17, 2021 ($7.80{\times}10^5$ N copies/L), followed by October 5, 2021 ($1.65{\times}10^5$ N copies/L)	[54]
23 November 2020–20 September 2021 24 February 2021 to 7 February 2022	Manholes or sewer cleanouts	U.S.A: San Diego	Affinity capture magnetic hydrogel particle	Amplification: qRT-PCR/ N1, N2 and E Sequencing: Illumina MiSeq / Superscript IV VILO (Thermo Fisher)	Sequencing: Delta (B.1.617.2) and its sublineages (AY.*), Omicron variant (B.1.1.529 and descendants), Epsilon and Alpha variants	[106]
August 2020–December 2020	Tap water	U.S.A: Florida, Miami-Dade Country	Ultracentrifugation, electronegative filtration	V2G-qPCR and qRT-PCR / N3 (CDC, US)	Daily average positivity rate of 23.6 cases per 1000 tested samples; average SARS-CoV-2 concentration was 1283 G.C./L	[184]
January–April 2020	Wastewater treatment plants	U.S.A: Louisiana	Ultrafiltration, electronegative membranes	qRT-PCR / N1, N2 (CDC, US)	13% (2/15) positivity rate; N1, N2 assays had a Geometric mean of SARS-CoV-2 concentration was $7.5{\times}10^3$ copies/L for N1 and $3.1{\times}10^3$ and $4.3{\times}10^3$ copies/L for N2.	[185]
August 26, 2020	Wastewater treatment plant, manhole	U.S.A: Tucson, Arizona	Vacuum filtration through membrane filters and ultrafiltration	Sequencing: Illumina NextSeq 500	B.1.5 sub-lineage	[186]
19 May 2020–15 July 2020	Wastewater interceptors	U.S.A: San Francisco	Ultrafiltration	Amplification: qRT-PCR/ N1 (CDC, US) Sequencing: NextSeq 550	N gene concentration ranged between ~2 and ~553 G.C./µl of RNA. Two adjacent SNVs (14222G and 14223C) were associated with variants from Washington State. Another two SNVs (8083A and 1738T) have been observed in four other states.	[24]
November 2020 to June 2021	Water reclamation facilities: influent wastewater	U.S.A: Nevada	Ultrafiltration	Amplification: qRT-PCR / N1, N2 (CDC, US) Sequencing: Illumina NextSeq, NextSeq 2000/ ARTIC v3	Amplification: Concentrations ranged between $3.00{\times}10^2$ to $9.28{\times}10^5$ G.C./L. Sequencing: B.1.617.2, B.1.1.7 (Delta variant); B.1.2., B.1.427/429 (Epsilon)	[187]
April 2022– June 2022	Sewage cleanout wastewater	U.S.A: California	Membrane filtration and centrifugation	(dd)RT-PCR/ N, S	Gene concentrations for SARS-CoV-2 RNA in solids ranged from ND to $4.4{\times}10^4$ copies/g (N) and from ND to $1.1{\times}10^5$ copies/g (S). Concentrations from liquid samples ranged	[188]

https://doi.org/10.3390/v18020205

					between ND to 41 copies/mL (N) and from ND to 6 copies/mL for (S)	
3 October 2023; 23–27 October 2023	Primary sludge from Regional Wastewater Facility, wastewater treatment plants	U.S.A: Santa Clara, San Jose, California	N.R.	RT-ddPCR/ N (Wolfe et al. (2021)	Batch 1 SARS-CoV-2 concentration ranged between 1 × 10^5 and 2 × 10^6 copies/ml^{-1}. Batch 2 SARS-CoV-2 concentration ranged between 1 × 10^6–2 × 10^6 copies/ml^{-1}.	[189]
27 August 2020–4 October 2020	Wastewater treatment plants	U.S.A: Hawaii, Honolulu	PEG precipitation	qRT-PCR / N1, N2 (CDC, US), E gene (Corman et al. 2020)	$10^{1.2}$-$10^{5.1}$ gene copies or G.C./L in liquid fraction,$10^{1.4}$-$10^{6.2}$ G.C./g in solid fractions	[190]
20–21 June and 11–12 July 2020	Wastewater treatment plants, pump station	U.S.A: Oregon	Electronegative membrane filtration	Amplification:RT-ddPCR/ N1, N2 (CDC, US) Sequencing: Illumina HiSeq3000/ Swift Biosciences primer set	Amplification: SARS-CoV-2 concentrations ranged between 2.92 to 5.13 Log_{10} G.C./L Sequencing: B.1.399, B.1, B.1.369, B.1.2, B.1.1.158	[72]
18 February –2 June 2020	Wastewater catchment areas	U.S.A.: 40 states	PEG precipitation, ultrafiltration	qRT-PCR / N1, N2 (CDC, US)	846 out of 1,751 samples were positive. In April, titers ranged between 10-100 copies/mL in 173 samples and were above 100 copies/mL in 78 samples. In May, 358 samples had titres between 10-100 copies/mL and 97 of samples had a concentration above 100 copies/mL 78 samples from 24 countries had viral titers higher than 10 copies /ml, 173 samples from other countries had between 10-100 copies/ml.	[191]
18–25 March 2020	Wastewater treatment plant	U.S.A: Massachusetts	PEG precipitation	Amplification: qRT-PCR / N1, N2 (CDC, US) and N3 (IDT) Sequencing: Sanger/ N1, N2, N3, S	Amplification: Mean SARS-CoV-2 titer estimated from all three primer sets ranged from 57 to 303 copies/ml Sequencing: Confirmation of identity match to the S gene (97-98%)	[192]
7 December 2020–9 May 2022	School manholes	U.S.A: Texas, Houston	HA filtration and bead beating	RT-ddPCR/ N1, N2 (CDC, US)	22.3% positivity rate (486/2176) for SARS-CoV-2; average RNA concentration was 141,248 copies/L	[193]
6 January–26 April 2021	Wastewater	U.S.A.: Carolina	Ultrafiltration	RT-ddPCR/ N1, N2 (CDC, US); VOCs: Alpha (B.1.1.7), Beta (B.1.351), Gamma (P.1), and Delta (B.1.617.2)	Positivity rate for SARS-CoV-2 RNA was 66.3% (189/285); Alpha, Beta and Gamma variants were detected	[194]
July and December 2020	Wastewater treatment plants	U.S.A: Carolina	Electronegative membrane filtration	RT-ddPCR, PCR (qRT-PCR)/ N1, N2 (CDC, US)	79% positivity rate for N1 and N2; RT-ddPCR concentration for plant 1:1.7×10^3 to 1.4×10^5 G.C./L (N1) and 2.4×10^3 to 5.9×10^4 G.C./L (N2); plant 2: 1.9×10^3 to 2.1×10^5 G.C./L (N1) and 1.9×10^3 to 2.8×10^5 G.C./L (N2)	[195]
May–October 2020	Wastewater treatment plants, sewer network	U.S.A: Hamilton County	Ultrafiltration	RT-ddPCR/ N1, N2 (CDC, US)	N1 and N2 concentrations across all samples were up to 10^5 copies/L	[196]
August 2020–May 2021	University campus wastewater	U.S.A: Florida, Gainesville	Electronegative adsorption	qRT-PCR / N1, N2 (CDC, US)	37% (235/ 628) positivity rate, N1 concentration range: 0–7.26 Log_{10} G.C./L (mean of 4.53); N2 concentration range: 0–6.77 Log_{10} G.C./L (mean of 4.40)	[197]

https://doi.org/10.3390/v18020205

March 2020–April 2021	Wastewater treatment facilities	U.S.A: Nevada	Hollow fiber ultrafiltration, centrifugal ultrafiltration	Amplification: qRT-PCR / N1, N2 (CDC, US), ORF1a (Lu et al. 2020) E Sarbeco (Corman et al. 2020) Sequencing: Illumina NextSeq 500/CleanPlex SARS-CoV-2 FLEX Panel	Amplification: Viral load ranged from 4.3 Log_{10} G.C./L to 8.7 Log_{10} G.C./L Sequencing: B.1.1.7 (Alpha) and B.1.429 (Epsilon) lineages	[198]
19 August–1 December 2020	Single buildings manholes, sewer cleanouts, municipal manholes	U.S.A: Louisiana, New Orleans	PEG precipitation	qRT-PCR / N1, N2 (CDC, US)	N1 and N2 genes were detected in 49.5% (53/107) of samples. N1 average concentration: 1.75×10^3 copies/100 mL; N2 average concentration: 6.76×10^3 copies/100 mL	[199]
November 2020–March 2022	Food processor facility's wastewater	U.S.A: Arizona, USA-Mexico border	Ultracentrifugation	qRT-PCR / N1 (CDC, US)	RNA was detected on 18 of 205 days in which wastewater was sampled and analyzed (8.8%). Highest concentration of N1 was 5.3 Log_{10} G.C./L	[200]
December 2020–October 2021	Catchment areas	U.S.A.: Michigan, Detroit metropolitan area	N.R.	RT-ddPCR/ N1, N2	N1 concentration: 1.92×10^2 G.C./L – 6.87×10^3 G.C./L; N2 concentration: 1.91×10^2 G.C./L – 6.45×10^3 G.C./L	[201]
1 October 2021–31 May 2022	Water Resource Recovery Facility	U.S.A.: Michigan, Detroit	Electropositive filtration (VIRADEL), PEG precipitation, polysulfone (PES) filtration	RT-ddPCR, qRT-PCR/ N1, N2 (CDC, US)	The mean concentrations for N1 and N2 genes in the filtered samples were 3.22E+04 and 1.50E+04 G.C./L, respectively. The average number of copies of the N1 and N2 genes in the VIRADEL samples was between 1.61E+03 and 1.63E+03 copies per liter. PEG samples had an average concentration of 1.61E+05 and 1.50E+05 copies/L for N1 and N2	[202]
November–December 2020	Wastewater treatment plants	U.S.A.: Charlotte region of North Carolina	Vacuum filtration	qRT-PCR, RT-ddPCR / N1, N2 (CDC, US)	Viral concentrations ranged between 10^3–10^5 copies/L for both RT-ddPCR and qRT-PCR; highest peak ranged between 1.15×10^5–1.96×10^5 copies/L	[203]
3 February–20 May, 2021	Sewage campus manholes	U.S.A.: Durham, North Carolina	Electronegative membrane filtration	qRT-PCR/ N1 (CDC, US)	Of the 114 tested samples, 32 (29%) had at least one positive qPCR replicate above the LOD. Technical replicates had typical values that ranged from 3 to 4 log_{10} G.C./sample	[204]
24 August–24 December 2020	Campus wastewater	U.S.A.: Syracuse (New York)	Ultracentrifugation	qPCR/ RdRp (IP2/ IP4, Institut Pasteur, Paris)	Out of 324 samples, 156 (48%) samples contained detectable levels of SARS-CoV-2 viral RNA material and 100 (31%) contained quantifiable levels of SARS-CoV-2 viral RNA material	[205]
November 2021–November 2022	Water reclamation facilities	U.S.A.: Nevada	Ultrafiltration	Amplification: qRT-PCR / N1, N2 (CDC) Sequencing: NextSeq 2000	Amplification: SARS-CoV-2 viral load ranged between the 4 G.C./µL (LOD) and 8.48×10^5 G.C./L (N1 gene) and 3.32×10^6 G.C./L (N2 gene) Sequencing: B.1.1.529, BA.2.12.1., BA.1, BA.1.1.16, BA.1.1.13, BA.1.1.12, BA.1.1.18, BA.2, BA.2.3, BA.3, BA.2.3.2, BA.2.12, BA.2.11, BA.5, BA 5.2.1, BA.5.10., BA.X, BQ (BQ.1	[106]

					and BQ.1.1), BF (BF.7) and BE (BE.1.1.1) variants were detected	
June 2020–June 2021	Wastewater reclamation facilities	U.S.A.: Georgia, Athens-Clarke County	N.R.	qRT-PCR / N1, N2 (CDC, US)	76% (193/254) positivity rate for influent samples; LOD was approximately 10^6–10^7 copies/ L; the maximum concentration was 2.12×10^7 copies/ L for N1 gene and 5.99×10^7 copies/L for N2 gene	[48]
1 February–21 May 2021	Manhole	U.S.A.	Vacuum filtration	qRT-PCR / N1, N2 (CDC, US)	N2 viral load was between 1.38×10^6 and 4.53×10^{11} (median of 1.29×10^9) G.C./day. N1 viral load ranged from 1.11×10^6 to 4.27×10^{11} (median of 1.04×10^9) G.C./day	[206]
October 2020–January 2021	Wastewater treatment plant	U.S.A.: Wisconsin	PEG precipitation	Amplification: qRT-PCR / N1, N2 (CDC, US), E (Corman et al. 2020) Sequencing: Illumina MiniSeq	Sequencing: 20A, 20B, 20C and 20G clades were detected	[207]
30 August 2020–20 January 2021	Wastewater treatment plant	U.S.A.: Wisconsin	Cellulose ester HA filters	RT-ddPCR/ N1, N2 (CDC, US)	For N1 concentrations ranged between 5086 and 14470 copies/L and for N2 concentrations varied between 1949 and 6878 copies/L	[50]
October–December 2020	Water reclamation facility, sewershed sub–catchments	U.S.A.: Washoe County, Nevada	Membrane filtration, ultrafiltration	qRT-PCR/ N1, N2	For November RNA concentrations in Reno and Sparks ranged from 2.62×10^4 to 2.16×10^6 G.C./L; In Sparks, concentrations ranged between 7.67×10^5 and 1.73×10^6 G.C./L until a decrease in early December	[208]
17 August, 2022–14 October, 2022	Wastewater treatment plant, manholes, sewer pump stations	U.S.A.: New Orleans, Louisiana	Magnetic hydrogel particles	Amplification:dPCR/ N1, N2 (CDC, US) Sequencing: Oxford Nanopore MinION /ARTIC nCoV-2019 v4.1 primers	Amplification: SARS-CoV-2 N1 and N2 genes had a positivity rate of 92 % and 97 %, respectively. Values for N1 ranged from 1.55×10^3 to 8.24×10^5 G.C./L (2.57×10^4 G.C./L average). For N2 values ranged from 1.55×10^3 to 1.02×10^6 G.C./L (2.77×10^4 G.C./L average) Sequencing: 22B Omicron variant was detected	[49]
September 2020–March 2021	Wastewater tank from prison facility	U.S.A.: New Orleans, Louisiana	Adsorption–extraction (Method A), adsorption–elution (Method B), electronegative membrane, ultrafiltration	qRT-PCR / N1, N2 (CDC, US)	The positivity rate using both methods was 33% (7/21). SARS-CoV-2 N1 and N2 genes had a positivity rate of 19.0% (4/21) and 23.8% (5/21), respectively for Method A. The positivity rates were 28.6% and 19.0% for N1 and N2, respectively, for method B. Concentration values for N1 ranged from 2.2–3.2 $\log_{10}$ G.C./L (Method A) and from 2.1–3.1 $\log_{10}$ G.C./L (Method B). For N2, values ranged from 1.9–2.5 $\log_{10}$ G.C./L (Method A) and 1.7–2.6 $\log_{10}$ G.C./L (Method B)	[209]
2 June–25 August 2020	Wastewater treatment plant	U.S.A.: Texas	Electronegative membrane	ddPCR/ N1, N2 (CDC, US)	77% SARS-CoV-2 positivity rate with concentrations ranging from 1.4×10^2 to	[210]

https://doi.org/10.3390/v18020205

					4.1×10^4 copies/L of wastewater	
6 October–7 October 2024	Dormitory wastewater samples	U.S.A.: Waco, Texas	Ultrafiltration	qRT-PCR / N2 (CDC, US)	Different viral loads were obtained depending on the extraction kit: Zymo Fecal Kit values ranging from 7300 to 17,000,000 copies/L; Zymo Quick-Viral RNA kit ranged from 9800 to 23,000,000 copies/L; New England BioLabs kit had the highest value (45,000,000 copies/L)	[211]
April–November 2021	Wastewater treatment plant	U.S.A.: San Antonio, Texas	Electronegative membrane	RT-ddPCR/ N1, N2 (CDC, US)	98.5% positivity rate for N1 and 93% for N2. Concentration ranged between 4.5×10^2 and 2.8×10^4 copies/L for N1 and 4.7×10^2 and 1.4×10^4 copies/L for N2.	[212]
28 April 2020–25 January 2022	Water treatment facilities	U.S.A.: Arkansas	PEG precipitation	Amplification: qRT-PCR/ ORF1ab, S, N (TaqMan™ 2019-nCoV Assay Kit v1) Sequencing: Illumina NextSeq 500/ Illumina SARS-CoV-2 Research Panel primer pools	Amplification: Wastewater influent viral load ranged from 4.70×10^2 copies/mL to 1.82×10^5 copies/mL Sequencing: 19A, 20A, 20C, 20G, 21A and/or 21J clades were detected	[213]
5 October 2020–19 September 2021	Wastewater treatment plant	U.S.A.: Ohio	Filtration, hollow fiber concentration, membrane filtration	qRT-PCR, ddPCR/ N1 and/or N2 (CDC, US)	SARS-CoV-2 raw viral load ranged from 1×10^1 to 1×10^5 G.C./L, with averages of 8.74×10^4 for N1 and 6.18×10^4 for N2 gene	[214]
September 2020–November 2022	Wastewater treatment plants	U.S.A.: Michigan	PEG precipitation	Amplification: ddPCR/ N1, N2 (CDC, US), N501Y, del69–70, T478K, L452R, K417N, N679K, Q954H, R408S, and A67V Sequencing: Oxford Nanopore MinION/ IDT ARTIC nCoV-2019 v3, v4 and v4.1 primer panel	Amplification: SARS-CoV-2 N gene concentration ranged from $7.2\text{x}10^2$ G.C./100 mL to $1.1\text{ x}10^6$ G.C./100 mL with a mean of $8.7\text{x}10^4$ G.C./100 mL; 20A, 20I, 21K, 21L, 22A, 22B clades were detected Sequencing: 19B, 20A, 20B, 20C, and 20 G, Alpha (20I), Delta (21I, 21 J) and Omicron (21 K, 21 L, 21 M, 22A, 22B, and 22C) variants were detected	[215]
April– December 2020	Septic tank, Pumping station, Hospital discharge	U.S.A.	Electronegative membrane filtration	RT-ddPCR/ N1, N2 (CDC, US)	SARS-CoV-2 RNA viral loads ranged from 5.79×10^3 to 3.96×10^5 G.C./L for N1 gene with a mean concentration of 6.61×10^4 copies/L. N2 concentration ranged between 1.40×10^3 and 2.34×10^5 copies/L	[216]
December 2021–July 2022	Wastewater treatment plants	U.S.A.: Las Vegas, Nevada	Hollow fiber ultrafiltration	Amplification: qRT-PCR / N1, N2 (CDC, US), E_Sarbeco (Corman et al. 2020), ORF1a (Lu et al. 2020) Sequencing: Illumina NextSeq 500/ CleanPlex SARS-CoV-2 FLEX Panel	Amplification: Concentrations ranged from 2.8 to 4.8 Log_{10} G.C./L Sequencing: Delta (AY.1 and AY.44), Alpha (B.1.1.7), Omicron (BA.1, BA.2, BA.2.12.1, BA.2.75, BA.4, BA.4.6, BA.5, BA.5.2) variants were detected	[217]
September 14, 2020–September 21, 2021	Sewage system, manholes, direct dispense from valve	U.S.A.: Tennessee	Ultrafiltration	qRT-PCR/N1 (CDC, US)	Viral load of SARS-CoV-2 RNA in raw sewage samples were $2.75\times10^2\pm5.49\times10^2$copies/L for residence halls, $1.97\times10^2\pm3.48\times10^2$copies/L for dormitories, $6.93\times10^2\pm2.12\times10^3$copies/L for fraternities, and	[218]

					$1.30\times10^2\pm1.91\times10^2$ copies/L for sororities	
3–16 August 2020; January 2021	Wastewater treatment facilities	U.S.A.: New England	PEG concentration	qRT-PCR, RT-ddPCR/ N1, N2 (CDC, US)	The average concentration for qRT-PCR was: 2.71±0.01 Log copies/mL (N1), 2.90±0.02 Log (N2) The average concentration for RT-ddPCR was: 2.89±0.02 Log copies/mL (N1), 2.81±0.01 Log copies/mL (N2)	[219]
November 2020–March 2021	Publicly owned treatment works	U.S.A.: San Francisco, California	Centrifugation	ddPCR/ N, S, ORF1a (ITD)	SARS-CoV-2 RNA gene viral loads ranged from 630 to 3.7×10^6 copies/g (N gene), non-detected (ND) to 3.2×10^6 copies/g (S gene), and ND to 3.0×10^6 (ORF1a) copies/g.	[220]
30 September 2020–23 March 2021	Wastewater treatment plant influent	U.S.A.: Shreveport, Louisiana	PEG precipitation	qRT-PCR / N1, N2 (CDC, US)	SARS-CoV-2 RNA positivity rate was 84.8% for N1 and 42.4% for N2 SARS-CoV-2 values were above the threshold of ~4800 G.C./L	[221]
March–October 2021	Manholes, pump station	U.S.A.: Seattle	PEG concentration	qRT-PCR / N1, N2 (CDC, US), E (Corman et al. 2020)	Concentrations ranged from 0.2 to 2.5 Log G.C./mL	[222]
January–February 2022	Manholes	U.S.A.: Atlanta	PEG centrifugation	RT-ddPCR/ N1, N2 (IDT)	For samples collected with swabs, the mean concentration was 3.7×10^2 copies/mL/day for N1 and 3.9×10^2 copies/mL/day for N2; for composite samples, the mean concentration was 5.2×10^1 copies/mL/day for N1 and 5.4 copies/mL/day for N2	[223]
September 2020–April 2021; May–December 2021	Wastewater treatment plant, sewer manhole	U.S.A.: Las Vegas, Nevada	N.R.	Amplification: qRT-PCR/ ORF1a, E, N1, N2 Sequencing: CleanPlex Illumina NextSeq 500/ SARS-CoV-2 FLEX Panel	Amplification: SARS-CoV-2 RNA concentrations ranged from 4.42×10^{14} to 3.72×10^{17} G.C./L Sequencing: Alpha, Epsilon, Omicron (BA.1) variants were detected	[224]
17 August, 2020–5 April, 2021	Wastewater treatment plant, sewer line, pump stations	U.S.A.: Kentucky	PEG precipitation	qRT-PCR/ N1	SARS-CoV-2 concentration ranged between 8 to 22707 copies/ mL. Solid sewage samples had a concentration of 2833 to 80 974 G.C./ g^{-1} dry weight	[225]
October 2021–January 2022	Wastewater treatment plants	Tunisia: Tunis	PEG precipitation	qRT-PCR/ E, S, N, and RdRP (Allplex™ SARS-CoV-2 Assay, Seegene)	Among 11 positive samples, the N gene (9/19) had a positivity rate of (47%), the S (8/19) and RdRp (8/19) genes had a similar detection rate (42%), and the E gene (5/19) detection was lower (21%)	[226]
December 2021 - July 2022	Wastewater treatment plants	Tunisia: Sidi Bouzid, Monastir, Gabes, Kairouan, Tunis, Beja	Electronegative membrane	Amplification: sdPCR / N1, N2 (CDC, US) Sequencing: Illumina NextSeq500/ Artic v4.1 primer sets	Amplification: 90% (27/30) of wastewater samples were positive with concentrations ranging between 5.3×10^3 and 9.02×10^6 copies/L. Sequencing: 85.7% of samples were Omicron (BA.1 + BA.2, BA.5) and samples collected in December 2021 were Delta	[227]
September–October 2020/ February–April 2021	Wastewater treatment plants	Tunisia: Monastir region	Adsorption-elution method	qRT-PCR/ N1, N2 (CDC, US); E, N and RdRp (Allplex 2019-nCoV kit	First sampling period: 61.11% (11/18) positivity rate; Second sampling period: 25.80% contained SARS-	[228]

				(Seegene, Seoul, South Korea)	CoV-2 RNA with viral loads between $0.18{\times}10^3$ copies/100 ml to $59{,}94{\times}10^3$ copies/100 ml	
22 October 2020–04 April 2021	Wastewater treatment plants	Canada: Ontario, Durham Region	Acetone precipitation	qRT-PCR/ N1 (CDC, US)	Linear standard curve varied between $1{\times}10^5$ and $1{\times}10^1$ copies/µL	[229]
2021 May–2021 October	Wastewater treatment plants	Canada: Calgary, Edmonton	Electronegative membrane	qRT-PCR /N1, N2 (CDC, US)	Concentration ranged between $2.4{\times}10^2$ to $2.9{\times}10^6$ copies per 100 mL	[230]
18 May 2020–7 June 2021	Wastewater treatment plants	Canada: Alberta	Ultrafiltration	qRT-PCR / N1, N2 (CDC, US), E (Qiu et al. 2021)	49.84% (918/1842) positivity rate	[231]
May 15, 2020–Oct 8, 2021	Wastewater treatment plants	Canada: Alberta	Centrifugation	qRT-PCR/ N1, N2 (CDC, US)	56.3% (1367/2430) positivity rate for SARS-CoV-2; for the first, second, third, and fourth waves the median concentrations were 109, 1100, 2784, and 4416 copies/100 mL	[232]
May 2020	Wastewater treatment plants	Canada: Calgary, Edmonton	Ultrafiltration	qRT-PCR / N1, N2 (CDC, US), RdRp, E (Pabbaraju et al. 2021)	N1, N2 and E were detected in all twelve non-frozen samples; among the 12 frozen samples N1 and N2 genes were found in ten samples and E gene was detected in nine samples	[233]
June 2020–May 2021	Wastewater treatment plants	Canada: Calgary	NaCl lysis and PVDF filter	qRT-PCR / N1, N2 (CDC, US)	98.06% (406/414) positivity rate	[108]
01 May 2022–01 July 2022	Wastewater treatment facility	Canada: Halifax, Nova Scotia	Electronegative membrane filtration	qRT-PCR / N1 (CDC, US)	SARS-CoV-2 viral load for composite samples was in the range of $2.0{\times}10^1$ to $2.1{\times}10^3$ G.C./mL; for passive sampling location values ranged between $1.0{\times}10^1$ to $1.1{\times}10^5$ G.C./mL	[234]
1 September–31 December 2021	Residence sewer lines, wastewater treatment facility	Canada: Halifax	Electronegative filtration	qRT-PCR/ N2 (CDC, US), Delta (B.1.617), Alpha (B.1.1.7), C28311T mutation (IDT, Coralville, IA, USA)	51 samples from the university residences and 20 WWTF samples were positive for SARS-CoV-2; C28311T (Omicron associated) mutation was detected. The mean SARS-CoV-2 concentration was 1.1×10^7 G.C./mL for Residence A, 3.0×10^6 G.C./mL for Residence B, and 1.3×10^6 G.C./mL for the WWTF samples.	[235]
5 August– 17 December, 2020	Hospital wastewater samples	Canada: Calgary	Filtration	qRT-PCR / N1, N2 (CDC, US), E (Corman et al. 2020)	SARS-CoV-2 N1-RNA had a median of 112 copies/ml	[236]
1 April–30 June 2020	Water resource recovery facilities	Canada: Ottawa, Gatineau	Filtration, PEG precipitation	qRT-PCR, RT-ddPCR/ N1, N2, N3, E_Sarbeco (Corman et al. 2020)	Concentrations ranged between $1.7{\times}10^3$ to $7.8{\times}10^4$ copies/L (Ottawa) and from $6.6{\times}10^4$ to $3.8{\times}10^5$ copies/L (Gatineau)	[90]
February-March 2021	Wastewater from a campus residence hall	Canada: Windsor-Essex	Hollow fiber ultrafiltration	qRT-PCR/ N1 (CDC, US), N gene containing D3L (Alpha specific mutation)	B.1.1.7 (Alpha) was detected	[237]
31 August 2020–21 March 2021	Wastewater treatment plant	Greece: Athens	PEG precipitation, Ultrafiltration	Amplification: qRT-PCR / N1, N2 (Water SARS-CoV-2 RT-PCR Test, IDEXX, USA) Sequencing: Ion Torrent PGM	Sequencing: Alpha (B.1.1.7); Beta (B.1.351); Gamma (P.1)	[131]
September 2020–November 2020	Wastewater treatment plant	Greece: Athens	PEG precipitation	Amplification: Nested RT-PCR/ N1 (CDC, US), Nsp3, Helicase, ORF3a Sequencing:	Positivity rate of 56.7% (17/30), D614G (S), P323L (RdRP) variants, Alpha (B.1.1.7)	[238]

				Ion OneTouch 2 System (Ion Torrent)		
02 December–06 May 2021	Wastewater Treatment Plant	Greece: Thessaloniki	Electronegative membranes	Amplification: qRT-PCR/ N2 (CDC, US) Sequencing: Illumina MiSeq, NextSeq500/ ARTIC V3 primers	Detected lineages: B.1, B.1.1, B.1.1.189, B.1.1.318, B.1.177, B.1.258, B.1.1.7, B.1.351	[239]
27 April 2020–2 December 2020	Wastewater treatment plant	Portugal	Hollow-fiber filters, PEG precipitation	qRT-PCR / E, N RdRp (Sarbecco)	97 samples were positive for at least one SARS-CoV-2 assay (97/204; 48%), at concentrations ranging from 10^3 to 10^6 G.C./L	[240]
July 2021–September 2021	Wastewater treatment plant, manhole, soil	Indonesia: Yogyakarta province	Electronegative membrane	qRT-PCR / N and ORF1ab (PerkinElmer, US)	54% (296/544) were positive for SARS-CoV-2 RNA, manhole samples presented the highest positivity rate (74%, 191/258 samples) and soil samples had the lowest positivity rate (3%, 2/60 samples)	[241]
27 July 2021–7 January 2022/ 18 January–3 June 2022	Wastewater treatment plants, manholes, soil	Indonesia: Yogyakarta province	Electronegative membrane	qRT-PCR/ N, ORF1ab (RT-PCR Assay (PerkinElmer®, USA)	During July 2021–January 2022, the highest positivity rate was 85.1%; during January– June 2022, the highest positivity rate was 94.4%; The highest concentration values were registered for N1 gene with 373.1 G.C. per reaction during Delta wave and 8909.8 G.C. per reaction during Omicron wave	[59]
12 February 2020–4 April 2020	Wastewater treatment plants	Spain: Valencia	Aluminium-driven flocculation	qRT-PCR/ N1, N2 (CDC, US)	12/12 samples were positive for SARS-CoV-2 with concentration ranging from 5.22 and 5.99 Log_{10} G.C./L.	[60]
April 2020–March 2021	Wastewater treatment plants	Spain: Valencia	Filtration and centrifugation	qRT-PCR/ N1, N2 (CDC, US)	The mean concentration of N1 was 3.48 ± 0.08 log10 G.C./L whereas for N2 the signal was slightly less intense 3.32 ± 0.08 log10 G.C./L	[242]
October 28, 2020–July 29, 2021	Sewage cleanouts, confluence sewer point	Spain	Adsorption, precipitation by aluminium	Amplification: qRT-PCR/ N1, E (Corman et al. 2020) Sequencing: Illumina MiSeq/ ARTIC V3 primers	The maximum levels of SARS-CoV-2 RNA was on November 3, (6.6×10^6 G.C./L)	[243]
12 March–14 April 2020	Wastewater treatment plants	Spain: Region of Murcia	Aluminum hydroxide adsorption-precipitation	qRT-PCR/ N1, N2, N3 (CDC)	83% (35/42) positivity rate for influent samples; 11% (2/18) positivity rate for secondary treated water samples; average titre of 5.4±0.2 Log_{10} G.C. /L; SARS-CoV-2 RNA titres for N1, N2 and N3 were of 5.1±0.3, 5.5±0.2, and 5.5±0.3 Log_{10} G.C./L, respectively.	[244]
28 September 2020–29 April 2021	Wastewater treatment plant, suburban pumping stations	Spain: Salamanca	Aluminum hydroxide adsorption-precipitation	qRT-PCR, RT-LAMP/ ORF1ab, E, N5, N15	According to the RT-LAMP analysis the maximum concentration was 1.64×10^4 G.C./μL^{-1} for ORF1ab and 1.51x10^2 G.C/μL^{-1} for the N15 region. For qRT-PCR the maximum was achieved with a concentration of 1.36×10^4 G.C/μL for N and 3.58× G.C/μL for ORF1ab	[245]
22 December 2020–26 March 2021	Nursing home sewage	Spain: A Coruña	Ultrafiltration	qRT-PCR/ N (TaqPath COVID-19 RT-PCR Kit)	61% positivity rate for SARS-CoV-2; the mean viral load between locations	[246]

					ranged between 798 and 3252233 copies/L	
7 July–7 August 2020	Wastewater treatment plant, sewage network	Spain: Castellón	Adsorption and precipitation by aluminium	Amplification: qRT-PCR/ N1, N2 (CDC, US), E (Charité, Germany) Sequencing: Illumina Miseq, Arctic V3 primers	Amplification: Concentrations ranged from 22387 to 645654 G/C/L for N1 and from 17378 to 162181 G/C/L for N2 across locations. Sequencing: D614G and A222V were detected and associated with the 20E/EU1 variant.	[247]
March–November 2020	Influent wastewater treatment plant	Spain: Catalonia	Ultrafiltration	qRT-PCR/ N1, N2 (CDC, US)	The highest viral load was 3.92×10^6 G.C./L (22nd of March)	[248]
8 July 2020–6 August 2020	Sewage treatment plants	India: Hyderabad Metropolitan City	Ultrafiltration	qRT-PCR/ E, N ORF1ab (Fosun COVID-19 RT-PCR Detection Kit)	Viral concentration varied between 661 copies/L and 24,469 copies/L	[249]
December 2020–January 2022	Influent wastewater pump station	South Africa: Cape Town	Homogenization and phase-separation	qRT-PCR/ N1, N2 (CDC, US)	The highest viral load was 66,483 copies/mL) and the median viral load was 639 copies/mL.	[250]
July–October 2020	Wastewater treatment plants	South Africa: KwaZulu-Natal	Ultrafiltration	ddPCR/ N2 (CDC)	SARS-CoV-2 concentration ranged from 648.1(±514.6) copies/mL to 1441.0(±1977.8)	[251]
1 June 2022–30 May 2023	Wastewater treatment facilities	South Africa: Buffalo	Centrifugation	qRT-PCR/ N1, N2 (CDC, US)	Concentrations ranged from 0.22×10^3 to 17.60×10^3 G.C./mL	[252]
January 2021–May 2022	Wastewater treatment plants	South Africa: Limpopo	Centrifugation	Amplification:qRT-PCR/ N (CDC, US), Alpha, Beta, Delta, and Omicron VoCs (TaqMan SARS-CoV-2 Mutation Panels) Sequencing: ATOPlex platform	Amplification: 75% (365/487) positivity rate for SARS-CoV-2. Sequencing: Delta (45%), Omicron (31.7%), Beta (5%) variants were detected. B.1, B.1.1, B.1.174, B.1.351, B.1.617, B.1.617.2, B.1.1.529, AY.45, BA.1, BA.2, BA.4, BE.1 (alias BA.5.3.1.1). Lineage AY.45 were found where AY.45 is associated with Delta variant and B.1.1.529 is associated with the Omicron variant.	[253]
May 2020–June 2022	Wastewater treatment plants and secondary sewer access points	United Arab Emirates: Abu Dhabi, Sharjah, Ajman, Umm Al Quwain, Ras Al Khaimah, Fujairah	Membrane Filtration	qRT-PCR/ RdRP (Primerdesign Ltd., UK, Cat#Z-Path-2019-nCoV) and RT-ddPCR/ N1, N2 (Bio-Rad, Cat#12013743)	The positivity rates were as follows: 59.46 % in AUD, 81.69 % in SHJ, 83.71 % in AJM, 93 % in UAQ, 81.76 % in RAK, and 81.16 % in FUJ. The median (G.C./L) was as follows: 205.6 in AUD, 916 % in SHJ, 1201.02 in AJM, 764.91 in RAK, 964.97 in FUJ, 1010.78 in UAQ	[254]
May–June 2020	Wastewater treatment plant, sewer access points	United Arab Emirates	Ultrafiltration, PEG precipitation	qRT-PCR/ RdRP (GENESIG COVID-19 kit)	Viral loads in samples taken from influents ranged from 7.50E+02 to over 3.40E+04 G.C./L; viral loads from untreated wastewater samples varied between 2.86E+02 and over 2.90E+04 G.C./L	[35]
May and June 2022	Wastewater treatment plant	Germany: Emschermündung, Dortmund-Scharnhorst, Dortmund-Deusen, Bottrop, Duisburg Alte Emscher, Dinslaken	Electronegative membrane	Amplification: qRT-PCR, ddPCR/ N1, N2 (CDC, US), L452R (Integrated DNA Technologies) SNP-PCR/ E484A/F486V (SARS-CoV-2	Sequencing: BA.4, BA.5 lineages were detected	[255]

				VirSNiP Mutations Assay) Sequencing: Ion Torrent		
-	Wastewater treatment plant	Germany: North-Rhine Westphalia	Electronegative membrane	Amplification: qRT-PCR/ N1, N2, K417N (Integrated DNA Technologies) qRT-PCR / N1, N2, Deletion 69/70, N501Y and K417N (Promega) Sequencing: Ion Torrent	Amplification: N1 and N2 genes had concentrations between 1E+13 and 1E+15 copies/day. Sequencing: Confirmation of Omicron variant presence	[256]
April 2020–March 2021	Sewage wastewater	Germany: Munich	Ultracentrifugation	Amplification: qRT-PCR and ddPCR/ N1 (CDC, US) Sequencing: Illumina HiSeq1500 (ARCTIC protocol v2)	Amplification: Viral load of SARS-CoV-2: between <7.5 and 3874/ml Sequencing: Detection of B.1.1.7, B 1.351, P.1 variants	[257]
10 January–29 May 2022	Wastewater treatment plant	Germany: Saxony	PEG precipitation	qRT-PCR / E, S (RealStar SARS-CoV-2 RT-PCR kit 1.0)	E gene had concentrations between 2.5×10^4 and 3.2×10^6 G.C./L at plant 1 and 1.3×10^4 and 3.1×10^6 G.C./L at plant 2	[258]
May 2021–July 2021	Wastewater treatment plants	Germany: Saxony	PEG precipitation	qRT-PCR, RT-ddPCR/ E (Corman et al 2020)	Of the 59 samples, 14 tested positive by qRT-PCR and 17 by RT-ddPCR	[259]
October–December 2021	Wastewater treatment plant, sub-sewersheds	Germany: Ruhr	Nanotrap® Magnetic Virus Particles	Amplification: qRT-PCR / N1, N2 (CDC, US) Sequencing: Ilumina MiSeq/ ARTIC primers	Sequencing: Delta (1.617.2), Mu (B.1.621), Eta (B.1.525), Lambda (C.37), Omicron (B.1.1.529, BA.2.75), Epsilon (B.1.427/B.1.429), Kappa (B.1.617.1)	[260]
December 2021–July 2023	Wastewater treatment plants	Germany: Berlin	N.R.	Amplification:dPCR/ N1, N2 (C DC, US) Sequencing: Illumina/ V4.1 ARTIC	The SARS-CoV-2 Omicron sublineage JN.1 (B.2.86.1.1) was identified along with XBB, EG.5.1, XBB.1.9, BA.2.86 variants	[261]
March 5, 2020–May 29, 2020	Wastewater treatment plant	Japan: Ishikawa, Toyama prefecture	PEG precipitation	qRT-PCR/ N2, N3 (CDC, US)	21 were positive according to at least one of the assays (N2, N3) and the concentration of quantifiable samples ranged between 1.2×10^4 and 3.5×10^4 copies/L	[262]
9 June 2020–19 August 2020	Wastewater treatment plants, manhole	Japan	Electronegative membrane, PEG precipitation, ultrafiltration, solid fraction	qRT-PCR / N1, N2 (CDC, US); N2 (Japanese National Institute of Infectious Disease- NIID)	1.6×10^2–1.3×10^4 G.C./L for the samples concentrated by solid fraction. The samples concentrated by other methods did not show consistent results.	[263]
13 September–07 October 2021	Wastewater treatment plants	Japan	PEG precipitation, Pegcision	qRT-PCR/ N1, N2 (CDC, US)	41% (11/27) positivity rate; Pegcision method: 4.7–5.0 Log copies/L; PEG precipitation: 4.5–4.8 Log copies/L	[264]
September 2020–January 2022, February–August 2022	Wastewater treatment plant	Japan: Yamanashi Prefecture	PEG precipitation	qRT-PCR / N1, N2 (CDC, US)	Positivity rate: 37 % between September 2020 to December 2021; 67% (88/132) during February–August 2022. Concentrations ranged from 3.5 to 5.0 Log_{10} copies/L (September 2020 to December 2021) and from 3.6 to 6.3 Log_{10} copies/L (January to August 2022)	[265]
August 2020– February 2021	Wastewater treatment plant	Japan: Miyagi, Sendai	Centrifugation	qRT-PCR / N1 (CDC, US)	18 (35.29%) samples tested positive; LOQ was around 1.61×10^2 copies/mL	[266]
January 2021–March 2022	Wastewater treatment plant	Japan: Yokkaichi City	PEG precipitation	qRT-PCR/ N1, N2 (CDC, US)	59% (38/64) positivity rate; among the 31 samples that tested positive for both	[267]

					regions the average concentration was 4.7±0.5 Log_{10} copies/L	
17 March–7 May 2020	River, Wastewater treatment plant: influent, secondary treated wastewater	Japan: Yamanashi Prefecture	Electronegative membrane-vortex, membrane adsorption, ultrafiltration	qRT-PCR/ N Sarbeco (Corman et al. 2020), N (NIID), N1, N2 (CDC, US)	Influent water viral load ranged between 4.0×10^3–8.2×10^4 copies/L while in secondary treated water viral load was 1.4×10^2–2.5×10^3 copies/L	[38]
1 November 2021–24 May 2022	Wastewater treatment plant	Japan: Sagami River basin	Coagulation (COPMAN/ EPISENS-S kit)	qRT-PCR/ N1 (CDC, US)	81/132 of the samples processed with COPMAN were positive; viral concentrations were up to 2.8×10^5 copies/L	[61]
01 July–19 October 2020	Wastewater treatment plant	Japan: Kanto region	PEG precipitation	qRT-PCR/ N1, N2 (CDC, US)	1 positive sample out of 34	[268]
4 March–8 July 2021	Wastewater treatment plants	Japan: Sapporo	EPISENS-S, PEG precipitation	qPCR/ N1 (CDC, US)	Positivity rate for WWTP A (EPISENS-S: 95 %, 16/19; PEG-QVR-qPCR: 0 %, 0/18); for WWTP B (EPISENS-S: 100 %, 18/18; PEG-QVR-qPCR: 0 %, 0/18); viral load for samples processed with EPISENS-S ranged from 3.55×10^2 to 1.32×10^4 copies/L and 1.32×10^2 to 3.89×10^4 copies/L for both WWTPs	[46]
24 February –27 October 2021	Influent wastewater	Japan: Kobe City	PEG precipitation	qRT-PCR/ N1, N2 (CDC, US)	RNA concentration in solid fraction and PEG-precipitated fraction ranged from 7.6×10^2 to 2.4×10^4 copies/L	[269]
16 April–14 June 2021	Wastewater treatment plants	Japan	EPISENS-S	qRT-PCR/ N1 (CDC, US)	97.8 % (269/275 positivity rate; maximum SARS-CoV-2 RNA concentration was 2.14×10^4 copies/L	[270]
22 November 2022–31 January 2023	Manholes from nursing facilities	Japan: Tokyo	N.R.	Amplification:qPCR/ N1 (CDC, US) Sequencing: Illumina MiSeq/ primers S012, S008, S013 and S009	Sequencing: Both BA.2.75 and BA.5 variants were detected	[271]
May–July 2020	Untreated wastewater (sewage)	Chile: Chillan	Membrane filtration and ultrafiltration	qRT-PCR / ORF1ab, N (TaqMan™ 2019-nCoV Assay Kit v1)	Average virus load of 7×10^3 G.C./L	[51]
2020 November–2022 February	Wastewater treatment tanks	Thailand: Bangkok	Centrifugation with glass beads	RT-PCR/ N, E, ORF1ab (Fosun COVID-19 RT-PCR Detection Kit) Sequencing: Illumina MiSeq/ ARTIC protocol v3 and v4 primers	Amplification: 102/ 215 samples were positive (42.5% positivity rate); LOD of the samples extracted from wastewater was 10^3 copies/mL for all tested genes Sequencing: Alpha, Delta and Omicron variants were detected	[73]
January–April 2021	Sewerage systems	Thailand: Bangkok	Electronegative membrane filtration	qRT-PCR/ N1, N2 (CDC, US)	Viral load for N1 gene ranged from 1.77 to 4.35 Log_{10} G.C./100 mL	[115]
January–February 2021	Hospital wastewater	Thailand: Samut Prakan province	Cellulose ester membrane filter	qRT-PCR/ ORF1ab, S, N (TaqMan™ 2019nCoV Assay Kit v1)	The following detection rates have been noticed: 76.5% for the N gene, 70.4% for the S gene, and 65.3% for the ORF1ab gene	[272]
April–July 2020	Wastewater treatment plants: influent, secondary sludge, and effluent	Mexico: Santiago de Queretaro	Ultrafiltration, adsorption	qRT-PCR/ N, S, RdRP (Sigma-Aldrich, USA)	Influent samples had a positivity rate of 36% and secondary sludge tested positive for 45% (for at least one of the targeted genes). The (LOD) was 3.6, 10 and 10 copies per qRT-PCR assay for RdRP, N and S genes	[273]
August 2020–January 2021	Wastewater treatment plant, groundwater	Mexico: Quintana Roo	Adsorption-elution, PEG precipitation, Sequential filtration	qRT-PCR System/ N1, N2 (CDC, US)	58% positivity rate for wastewater samples.	[274]

					1.8×10^3 to 7.5×10^3 G.C./L^{-1} for N1 and 2.4×10^2 to 5.9×10^3 G.C. /L^{-1} for N2	
29 June & 6 July 2020	Wastewater treatment plants, open-air channels	Mexico: Hidalgo state	Membrane filtering, PEG precipitation	qRT-PCR/ N1, N2, N3 (CDC, US)	On 29 June concentrations varied between 3.0×10^4 and 1.97×10^5 copies/L On 6 July concentrations varied between 8.15×10^3 and 2.98×10^4 copies/L	[275]
20 June–20 December 2020	Wastewater treatment plants, hospital effluent wastewater	Mexico: Monterrey Metropolitan Area	N.R.	qRT-PCR/ N1, N2 (CDC, US)	Concentration in positive samples was between 1.2×10^3 and 3.5×10^6 G.C./L	[55]
April 2020–February 2021	Sewer catchments	Mexico: Monterrey Metropolitan area	Ultrafiltration	qRT-PCR/ E (Charité, Germany)	Before the first wave the peak concentrations were 1.6×10^6 and 1.5×10^6 G.C./L; before the second wave the peak concentrations were 1.0×10^{13} to 3.3×10^{14} G.C./L; the peak before 5 January 2021 was 3.5×10^{14} G.C./L.	[276]
January–December 2021	Wastewater treatment plant	Egypt: Giza	PEG precipitation	RT-PCR/ E, S, or N (Park et al. 2020); qRT-PCR/ N1, N2 (CDC, US)	Samples tested with qRT-PCR showed 62.5% (30/48 samples) positivity for SARS-CoV-2	[277]
November 2020–October 2021	Wastewater treatment plant	Iran: Tehran	Filtration	qRT-PCR / ORF1ab, N (Phonix Biotech Inc)	5 samples out of 12 were positive	[278]
September 2020–April 2021	Wastewater treatment plant	Iran: Tehran	PEG precipitation	qRT-PCR/ ORF1ab, N (TaqPath™ Covid-19 RT-PCR Kit)	100% (91/91) positivity rate for N assay and 81% (74/91) for ORF1ab assay; viral loads ranged from 40 to 45,000 G.C./L	[81]
November 2020–May 2021	Sewage pipe network	Iran: Tehran	PEG concentration	qRT-PCR/ N (CDC, US), ORF1ab (Sansure Biotech, China)	34/34 (100%) of samples were positive for at least one of the targeted genes Composite samples: 67% (23/34) positivity rate Grab samples: 47% (16/34) for both targeted genes	[279]
22 October–20 November 2021	Wastewater drain system	Bangladesh: Noakhali district	PEG precipitation	Amplification:qRT-PCR/ ORF1ab, N gene (Sansure Biotech Inc., China)	Amplification: 47 % (57/120) of wastewater samples were positive for N1 and ORF1ab; Sequencing: Delta (B.1.617.2)	[280]
September 2020–January 2021	Hospital wastewater (direct pipes, septic tank/ABR inlets)	Bangladesh: Dhaka	Calcium flocculation-citrate dissolution	qRT-PCR/ ORF 1ab, N (Novel Coronavirus (2019-nCoV) Nucleic Acid Diagnostic Kit)	67 % (60/90) positivity rate; the median concentration for SARS-CoV-2 was 141 GEC/mL (range: 13–18,214)	[281]
August 2020 –May 2021	Drains, canals, hospital septic tanks, pumping stations, wastewater treatment plan	Bangladesh: Dhaka	Calcium flocculation-citrate dissolution	Amplification: qRT-PCR/ ORF1ab, N Sequencing: Oxford Nanopore MinION/ ARTIC nCoV-2019 protocol v3	Median Log_{10} concentration: 5.2 for N (range 3.9–7.6); 4.9 G.C./L for ORF1ab (range 3.96–7.3); Sequencing: Clades: 20A, 20B, 21A, 21J; Pango lineages: B.1, B.1.1, B.1.1.25, and B.1.617.2	[282]
July 2020–August 2021	Community discharges of domestic wastewater	India: Greater Hyderabad, Telangana	Ultrafiltration	qRT-PCR/ E, N, ORF1ab, (Fosum RT-PCR Kit)	Monthly collected samples showed values between 2036 RNA copies/L (January 2021) and 61,160 RNA copies/L (November 2020)	[283]
5–11 September 2020	Sewage treatment plants (STP), sewage pumping station (SPS)	India: Chennai	Composite (COM), supernatant (SUP), sediment (SED) and syringe filtration (SYR)	qRT-PCR/ N1, N2 (CDC, US)	Samples processed with SED had higher viral loads: for STPs ranged between 9.66×10^4 and 1.99×10^5 G.C./L; for SPSs ranged from 1.41×10^4 to 9.96×10^4 G.C./L	[284]
7 August–30 September 2020	Wastewater treatment plants	India: Gandhinagar	Filtration, PEG precipitation	qRT-PCR/ S, N, ORFab (TaqPathTM	40 out of 43 samples were positive for at least 2 genes; the average viral	[285]

				Covid-19 RT-PCR Kit)	load for each gene were the following: ~1223 copies/L (S-gene), followed by ~1022 copies/L (N-gene) and~485 copies/L (ORF1ab-gene)	
8 and 27 May 2020	Wastewater treatment plants	India: Ahmedabad, Gujarat	PEG precipitation	qRT-PCR/ ORF1ab, N, S (TaqPath™ Covid-19 RT-PCR Kit)	100% positivity rate; concentration ranged between 5.6×10 copies/L and 3.5×10^2 copies/L	[286]
May–August 2020	Wastewater treatment facilities	India: Jaipur, Rishikesh, Haridwar, and Roorkee	Vacuum filtration and centrifugation	qRT-PCR/ E, N, RdRP (Allplex™ 2019-nCoV kit)	33.3% positivity rate for SARS-CoV-2; 3/40 samples were positive in Jaipur; 5/14 samples were positive in Haridwar district.	[287]
7 April–10 June 2021	Wastewater treatment plants: influent and effluent	India: Mumbai	PEG precipitation	qRT-PCR/ N, ORF1b-nsp14, RdRp (TRUPCR SARS-CoV-2 RT-qPCR Kit)	76% positivity rate for SARS-CoV-2; The highest recorded concentration for the N gene was found in the raw wastewater of Bhandup WWTP with a value of 4.27 ± 1.62 Log_{10} G.C./100 mL, while for ORF1b was 4.43 ± 1.66 Log_{10} G.C./100 mL, in the same location. For RdRp, the highest recorded value was 3.79 ± 2.67 Log_{10} G.C./100 mL, in the raw wastewater of Charkop.	[288]
July 2020–February 2021	Wastewater treatment plants, a hospital, sewer lines, and rivers	Nepal: Kathmandu Valley	Electronegative membrane, ultrafiltration	qRT-PCR/ N (Sarbeco), N (NIID), N1, N2 (CDC, US)	Positivity rate for total treated effluents of WWTPs was 47% (16/34). Highest concentration was recorded at WWTP A (5.5±1.0 Log_{10} G.C./L) by the N_Sarbeco assay.	[289]
18 October–08 December 2023	Influent points of sewer ponds and treatment plants	Zambia: Copperbelt, Eastern provinces	Skimmed milk flocculation, bag-mediated filtration, PEG precipitation	Amplification: qRT-PCR, ORF1ab, N, S (TaqPath COVID-19 CE-IVD qRT-PCR kit mix) Sequencing: Illumina NextSeq 2000/ ARCTIC V4 primers	Amplification: 62 (40%) of 155 samples tested positive for SARS-CoV-2 Sequencing: Omicron (BA.1, BA.2, BA.3, BA.4, BA.5, BA.2.86, BA.1.1.45, JN.1., JC.1, JC.2, JC.6, XBB.1.45., XBB.2.9, GE.1.4, GE.1.3, GE.1.5, XM)	[290]
February–November 2023	Wastewater treatment plants	Ethiopia: Addis Ababa	–	qRT-PCR/ ORF1ab, N, S (TaqPath™ COVID-19 CE-IVD RT-PCR kit)	SARS-CoV-2 was detected in 94% of samples; Median viral concentration (G.C./L): Kality - 60,388 (21544–430,339), 26,355 (7,748–125,372) and 6,2,573 (12,221–24,9,039) for ORF1ab, N gene, and S gene, respectively Bulbula - 52,780 (19,078–375,512), 38,301 (12,273–186,201) for ORF1ab, N gene, and S gene, respectively Mikililand - 64,762 (18087–309,415), 45,580 (15,681–158,475), and 51,454 (11,318–184,333) for ORF1ab, N gene, and S gene, respectively	[291]
25 October 2020–13 December 2020/ 25 December 2021–9 January 2022	Sewage	Ethiopia: Addis Ababa	Ultracentrifugation	RT-PCR/ ORF1ab, E	Between October–December 2020 positivity rate was 9%; between 25 December 2021 and 9 January 2022 positivity rate was 88.9%	[292]

November 2020–November 2022	Raw sewage and wastewater	Ghana: 7 regions	PEG precipitation	Amplification: RT-PCR/ VERI-Q nCoV-OM detection kit Sequencing: Oxford Nanopore's MinION Sequencing/ LunaScript RT SuperMix	Amplification: 59 (17%) tested positive for SARS-CoV-2 Sequencing: Alpha (B.1.1.7) and Delta (AY.36)	[293]
February–May 2021	Wastewater treatment plant: influent (untreated), effluent (treated) and returned activated sludge	Malaysia: Kuala Lumpur and Selangor	PEG precipitation	qRT-PCR/ E, RdRp (Corman et al. 2020)	66% positivity rate (4/6) where E gene showed values between 1.7×10^5 – 1.0×10^7 copies/L and	[47]
2 August 2020–20 November 2020	Wastewater treatment plants	Belgium	Ultrafiltration, PEG precipitation	qRT-PCR, RT-dPCR/ N1, N2 (CDC) E-gene (Corman et al. 2020)	qRT-PCR concentrations ranges (copies/µL): N1- 0.08-0.46; N2- 0.10-0.74; E- 1.27- 13.57; RT-dPCR: N1-0.13-1.77, N2-0.53-1.00, E-0.13-0.27	[294]
8 November–12 December 2020	Sewer pipes	Philippines: Davao	PEG precipitation	Amplification:qRT-PCR/ E, N, RdRP (AllplexTM 2019-nCoV) Sequencing: MinION Mk1B device/ ARTIC nCoV-2019	Amplification: 91.7% positivity rate (22/24) with concentrations ranging from 7.8 to 40.2 ng/uL Sequencing: 17 previously unreported non-synonymous SNPs were detected in the coding regions of ORF1a, ORF1b, ORF6, ORF7a, ORF8, and N	[295]
July 2021–September 2022	Wastewater treatment plant	Latvia: Liepaja, Ventspils, Jurmala, Jelgava, Riga	PEG precipitation	Amplification: qRT-PCR/ N1, N2 (CDC, US) E Sarbeco (Corman et al. 2020); Sequencing: Illumina MiSeq/ SCoV2-RBD-2-i5Fw, SCoV2-RBD-2-i7Rs primer pairs	Delta and Omicron variants were detected (BA.1, BA.2, BA.4/5)	[296]
July–August 2021	Wastewater treatment plant	Argentina: Santa Fe, San Justo City	Ultrafiltration	qRT-PCR/ ORF1ab, N (PerkinElmer®)	30% positivity rate; average number of genetic copies/100 mL: location S1 had 139,581, S2 had 57,256 and S3 had 0 for N1 gene; location S1 had 80,890 for ORF1ab gene but in location S2 and S3 the virus was not detected.	[397]
13 August–30 December 2020	Sewer manholes	Argentina: Salta city	PEG precipitation	qRT-PCR/ N1 (CDC, US)	SARS-CoV-2 viral load ranged between 1.77×10^4 and 4.35×10^7 G.C./L	[398]
22 July 2020– 30 November, 2020	Wastewater treatment plants	Argentina: Mendoza province	PEG precipitation, PAC flocculation	qRT-PCR/ N1, N2 (CDC, US)	Highest values for both genes were registered during September 14–20, 2020 where N1 = 13,240 and N2 = 21,138 (copies/100 mL) at El Paramillo WWTP; N2 values peaked on September 7-13 at Campo Espejo WWTP 2020 with a concentration of 30995 (copies/100 mL)	[299]
May 2020–August 2021	Sewage network	Argentina: Córdoba province	PEG precipitation	RT-PCR/ N, ORF1ab (DisCoVery SARS-CoV-2 RT-PCR Detection Kit), E (LightMix® Modular SARS and Wuhan CoV E-gene kit)	43.4% (86/198) positivity rate for DisCoVery SARS-CoV-2 RT-PCR Detection Kit and; 51.5% (102/198) positivity rate for LightMix® Modular SARS and Wuhan CoV E-gene kit and 71.2% (141/198) detection rate for both kits	[300]
8 November 2021–28 February 2022	Wastewater treatment plant	Finland: Helsinki	Ultrafiltration	Sequencing: Illumina	Detected variants: Delta (B.1.617.2), Omicron (BA.1), Omicron (BA.2)	[301]

				NovaSeq 6000/ ARTIC.v4, ARTIC.v4.1 primers		
August 2020–May 2021	Wastewater treatment plant	Finland: 27 cities	Ultrafiltration	qRT-PCR/ N2 (CDC, US), E_Sarbeco (Corman et al. 2020)	Two cities with a small population exhibited the highest mean SARS-CoV-2 RNA copy numbers in WW: 7.72±0.23 (Vihti) and 7.89±0.40 Log_{10} (Salo) G.C./day/person.	[302]
21 February 2021–11 December 2022	Wastewater treatment plants	Finland: Helsinki, Espoo, Turku, Oulu, Tampere, Kuopio, Rovaniemi, Lappeenranta and others	Ultrafiltration	qRT-PCR/ N1, N2 (CDC, US)	SARS-CoV-2 RNA was detected more frequently using the N2 assay than the N1 assay. In Laboratory 1, it was detected in 91.5% of samples, and in Laboratory 2, it was detected in 87.4% of samples. In Laboratory 2 only, it was detected in 76.6% of samples using the N1 assay.	[303]
July 2020– May 2021	Sewer network	Czech Republic: Prague	PEG precipitation	qRT-PCR/ N1 (CDC, US), S (JRC), RdRp (Corman et al. 2020)	46% positivity rate; numbers for N1 viral load in the positive samples ranged from 10^1 to 10^7 G.C./L of wastewater	[304]
October– December 2021	School wastewater	Czech Republic: Prague	Homogenization	qRT-PCR/ N1 (CDC, US), S (JRC), RdRp (Corman et al. 2020)	Positivity rate for all schools was 50.4 %, and increased to 60.1 % when borderline samples were taken into consideration	[305]
May 2020–February 2023	Wastewater treatment plant	Czech Republic: Moravia, Bohemia	PEG precipitation	qRT-PCR/ N, Nsp12	71% positivity rate; viral load in wastewater had increased from 10^3 to 10^5 SARS-CoV-2 genome equivalent/day/person	[306]
10 June 2021–17 January 2022	Sewage catchment areas	Pakistan: Karachi	Filtration, skimmed milk flocculation	Amplification: qRT-PCR/ N1, N2, E (SARS-CoV-2 Wastewater qRT-PCR Promega kit) Sequencing: Illumina iSeq100, MiSeq/ ARTIC-NEB V3 and V4	Amplification: 123 out of 151 samples (81.5%) tested positive for N1, N2 or E genes Sequencing: Iota, Eta, Beta, Alpha, Delta (predominant) and Omicron variant	[307]
March–April 2020	Sewage	Pakistan	PEG precipitation	qRT-PCR/ ORF1ab (RT-PCR Kit for detecting 2019-nCoV by BGI China), ORF1ab, N (qRT-PCR for Novel Coronavirus (2019-nCoV) Nucleic Acid Diagnostic Kit)	27% (21/78) positivity rate for SARS-CoV-2 RNA	[308]
19 July–27 September 2023	Sewage collectors, wastewater treatment plant	Romania: Bucharest	Ultrafiltration	Amplification:dPCR/N1, N2 (CDC, US) Sequencing: Illumina Miseq/ Illumina COVIDSeq RUO protocol	Amplification: 72.7% positivity rate with a concentration range from 3.36 copies/L Log_{10} to 4.74 copies/L Log_{10} Sequencing: Clades: 23D, 23B, 23E Pango lineages: XBB.1.9.1, XBB.1.16.1, XBB.16, XBB.2.3	[19]
January 2020, October, November, and December 2020, January 2021	Sewage water	Romania	N.R.	RT-PCR/ E, RdPR, N (Allplex 2019-nCoV assay, Seegene)	The Allplex 2019-nCoV assay detected all three SARS-CoV-2 genes (E, RdPR, and N) in one sample from Siret. Two genes (E and RdPR) were detected in one sample, while RdPR and N were detected in four samples, RdPR in five, and N in one.	[309]
1–15 June 2020	Untreated hospital wastewater	Slovenia: Ljubljana	Glass fiber filtration, ultrafiltration	qRT-PCR/ E, RdRP (Corman et al. 2020)	66.7% (10/15) positivity rate for at least one SARS-CoV-2 target.	[310]

https://doi.org/10.3390/v18020205

June–January 2022	Wastewater treatment plant, sewer system	Singapore	Ultrafiltration	qRT-PCR/ N (CDC, US), ORF1ab, S	Positivity rate for samples collected from WWTPs: 41.23% (416/1009). Positivity rate for samples collected from sewage: 5.50% (11/200). Viral loads for positive samples varied between 2.48–5.35 Log_{10} G.C./L	[311]
07 January–04 February 2022	Sewersheds	Morocco: Agadir and Inezgane	PEG/NaCl precipitation	Amplification: qRT-PCR/ N, ORF1ab (TaqPath™ COVID-19 CE-IVD RT-PCR Kit) Sequencing: Ion Torrent/ Ion AmpliSeq™ SARS-CoV-2 Insight Research Panel	Amplification: In Agadir city values ranged between $8.2932\times10^4\pm1.5479\times10^4$ and $5.07865\times10^5\pm5.8759\times10^4$ copies/L In Inezgane city values ranged from $6.1244\times10^4\pm8.552\times10^3$ to $4.97506\times10^5 \pm 1.8061\times10^4$ copies/L Sequencing: BA.1 and BA.2 were detected	[312]
June 2020– November 2020	Wastewater treatment plants, hospital sewage	Hungary: Budapest	Flocculation, ultrafiltration	qRT-PCR/ N1 (CDC, US)	The concentration range was up to 7.14×10^5 G.C./L)	[313]
October 2022– January 2023	Wastewater treatment plants	Qatar	PEG precipitation	Amplification: qRT-PCR/ N1, N2 (CDC, US) Sequencing: Illumina Nextseq2000/ Artic V5.3.2	Amplification: The highest noted viral load was $2.35\text{x}10^{15}$ copies/day Sequencing: Omicron (XBB.1, XBB.2) was detected	[314]
February–August 2021	Wastewater treatment plant	Qatar	PEG precipitation	Amplification: qRT-PCR/ N1, N2 (CDC, US) Sequencing: Illumina Miseq/ CleanPlex SARS-CoV-2 Panel	Amplification: The maximum viral was observed on 21st March 2021 with a value of 1.15×10^{15} copy/day. The lowest load was noticed on 20th June 2021 with a value of $5.70 \text{ x } 10^{11}$ copies/day. Sequencing: B.1.1.7 (Alpha variant) and B.1.351 (Beta variant) were detected	[315]
15 April–9 July, 2020	Wastewater treatment plant	Saudi Arabia: Jeddah	Electronegative membrane filtration	qRT-PCR/ N1, N2, N3 (Forster et al. 2020)	75.4% (43/57) positivity rate for SARS-CoV-2 where: 54.4% (31/57) for N1, 45.6% (26/57) for N2 and 40.4% (23/57) for N3	[316]
August 2020–September 2022	Wastewater treatment plant	Saudi Arabia	Electronegative membrane filtration	Amplification: qRT-PCR/ N1, N2 (CDC) Sequencing: GridION/ ARTIC-V3 protocol	Amplification: Concentration ranged between 216 and 3250.7 copies/L Sequencing: AY.4.2, AY.1 and B.1.617.2	[317]
February 2020–October 2021	Sewage treatment plant, septic tankers	Bahrain: Muharraq	PEG precipitation	qRT-PCR/ N1, N2, E (Promega Corporation, USA)	84.62% (55/65) positivity rate; concentrations ranged between 0 and 11,508 RNA copies/mL for samples from the sewage treatment plant and from 27 to 19,105 copies/mL for samples collected from the septic tanks from quarantine facilities	[318]
1 March 2020–December 2020	River water (Danube)	Serbia: Belgrade	Ultrafiltration	qRT-PCR/ N1, N2 genes (CDC, US), E gene (Sarbeco) (Corman et al. 2020)	SARS-CoV-2 copy number ranged from 5.96×10^3 up to 1.30×10^4 G.C./L.	[62]
December 2020, February–March 2021	Wastewater treatment plants	Antarctica	Skimmed milk flocculation	Amplification: qRT-PCR/ N1, N2, E, RdRp (Taqman® Fast	Amplification: 60% (12/20) positivity The first positive sample had a viral load of 121,600	[319]

https://doi.org/10.3390/v18020205

	Viral 1-Step Master Mix)<br>Sequencing:<br>Next Seq500/ ARTIC V3	G.C./L (influent) and 14,670 G.C./L (effluent).<br>Sequencing:<br>B.1.1.451, B.1.1, B.1.1.409 lineages were detected	

N.R. – Not reported

https://doi.org/10.3390/v18020205
